# *Eimeria* spp. in Cattle: A Global Systematic Review and Meta‐Analysis

**DOI:** 10.1002/vms3.70991

**Published:** 2026-05-11

**Authors:** Laya Shamsi, Ali Pouryousef, Mohammad Reza Mohammadi, Mostafa Omidian, Milad Badri, Oskar Nowak, Ali Asghari

**Affiliations:** ^1^ Department of Veterinary Pathobiology SR.C., Islamic Azad University Tehran Iran; ^2^ Leishmaniasis Research Center Sabzevar University of Medical Sciences Sabzevar Iran; ^3^ Department of Bacteriology Faculty of Medical Sciences Tarbiat Modares University Tehran Iran; ^4^ Skin Diseases and Leishmaniasis Research Center Isfahan University of Medical Sciences Isfahan Iran; ^5^ Medical Microbiology Research Center Qazvin University of Medical Sciences Qazvin Iran; ^6^ Institute of Human Biology and Evolution Faculty of Biology Adam Mickiewicz University Poznan Poland; ^7^ Department of Basic Medical Sciences Khoy University of Medical Sciences Khoy Iran

**Keywords:** cattle, coccidiosis, *Eimeria* spp, prevalence, risk factors, species, systematic review

## Abstract

*Eimeria* spp. are major protozoan parasites of cattle, causing coccidiosis with substantial economic and animal health impacts worldwide. This study systematically reviewed and meta‐analysed the global prevalence, species distribution and associated risk factors of *Eimeria* spp. in cattle. Various international databases were searched from inception to 16 April 2025. Eligible studies reported extractable prevalence data for naturally infected cattle. Pooled prevalence was estimated using a random‐effects model, with heterogeneity assessed by *I* ^2^ statistic. Subgroup analyses were conducted by publication year, continent, country and sample size. Age‐ and sex‐specific data were analysed descriptively due to missing denominators. Genetic diversity and seasonal patterns were summarized descriptively. Meta‐regression evaluated sample size, annual precipitation, publication year and national cattle population. Sensitivity analysis and funnel plot (Egger's test) assessed robustness and publication bias. A total of 203 studies including 133,740 cattle from 55 countries were analysed. The global pooled prevalence of *Eimeria* spp. in cattle was 33.6% (95% confidence interval [CI]: 29.6%–37.8%), with substantial heterogeneity (*I* ^2^ = 99.4%). Prevalence ranged from 27.1% (2012–2018) to 40.8% (≤2011), 29.5% in Asia to 67.4% in Central America and 1% (Macedonia) to 94.2% (Costa Rica), though some national estimates were based on single studies. Calves <1 year accounted for the highest proportion of positives (56.3%, 95% CI: 46.2–65.8), and females showed higher infection rates (66.7%, 95% CI: 61.7–71.4). Infections peaked during warm and humid periods. Sixteen *Eimeria* species were identified in cattle; *E. bovis* and *E. zuernii* predominated, followed by *E. auburnensis*, *E. ellipsoidalis*, *E. cylindrica* and *E. alabamensis*. Sensitivity analyses confirmed estimate stability. Meta‐regression identified sample size as the only significant predictor, explaining 4% of heterogeneity. Publication bias was detected (*p* < 0.05). *Eimeria* infection imposes a substantial global burden in cattle, particularly among calves and females. Although sample size influenced reported prevalence, marked heterogeneity persists. Standardized reporting and geographically balanced studies are needed to better inform global coccidiosis control strategies.

## Graphical abstract

This global synthesis, based on 203 studies across 55 countries, revealed a substantial worldwide burden of *Eimeria* spp. infection in cattle (33.6%), with calves and females showing disproportionately higher infection frequencies and *E. bovis* and *E. zuernii* identified as the predominant pathogenic species. Furthermore, the high heterogeneity observed across studies highlights the need for standardized methodologies and improved geographic representation to better support targeted coccidiosis control strategies.

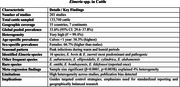

## 1 Introduction

*Eimeria* spp. (Apicomplexa: Coccidiasina) are obligate intracellular protozoa with a direct life cycle, in which ingestion of sporulated oocysts leads to intestinal invasion, replication and shedding of new oocysts into the environment (Burrell et al. 2020; López‐Osorio et al. 2020). Over 1700 species have been described in vertebrates (Bangoura et al. 2022), and more than 12 occur in cattle (Disfani et al. 2025).

Coccidiosis due to *Eimeria* spp. is a major parasitic disease in livestock and poultry, causing intestinal pathology, reduced performance and substantial economic losses (Chartier and Paraud 2012; Mesa‐Pineda et al. 2021). In cattle, clinical disease is particularly important in calves, with *Eimeria bovis* and *Eimeria zuernii* recognized as the most pathogenic species (Daugschies and Najdrowski 2005). These parasites damage the distal intestine and colon, leading to diarrhoea, dehydration, weight loss and mortality, whereas adults often act as asymptomatic carriers that sustain environmental contamination (Daugschies and Najdrowski 2005; von Samson‐Himmelstjerna et al. 2006).

Globally, bovine coccidiosis ranks among the most economically important parasitic infections, reducing growth rates, feed efficiency and productivity, while increasing veterinary costs (Daugschies and Najdrowski 2005; Pal and Tariku 2024). Although many regional surveys have investigated prevalence and risk factors, no comprehensive synthesis of global data has been available. Therefore, this study conducted a systematic review and meta‐analysis of published evidence up to April 2025 to estimate the worldwide prevalence of *Eimeria* spp. in cattle, identify associated risk factors and describe species distribution. These findings will support more effective control strategies and help reduce the economic burden of coccidiosis in cattle production systems.

## 2 Methods

### 2.1 Study Design and Methodology

This study was designed as a systematic review and meta‐analysis to estimate the global prevalence of *Eimeria* spp. in cattle and to identify associated risk factors. The methodology followed the Preferred Reporting Items for Systematic Reviews and Meta‐Analyses (PRISMA) guidelines to ensure transparency, reproducibility and methodological rigour (Page et al. 2021).

### 2.2 Literature Search Strategy

A comprehensive and structured literature search was conducted in the electronic databases PubMed, Scopus and Web of Science for articles published from inception up to 16 April 2025. In addition to major databases, Google Scholar was searched to identify studies that might be missing from conventional databases, thereby minimizing the risk of overlooking potentially relevant literature. The search terms used included a combination of Medical Subject Headings (MeSH) and free‐text keywords such as: ‘Eimeria’, ‘coccidiosis’, ‘intestinal parasites’, ‘species’, ‘cattle’, ‘ruminants’, ‘livestock’ and ‘prevalence’. Boolean operators (AND, OR) were used to combine keywords appropriately. Reference lists of relevant articles were also manually screened to identify additional eligible studies.

### 2.3 Eligibility Criteria

Studies were included if they met the following criteria: (1) reported the prevalence of *Eimeria* spp. in cattle, (2) provided sufficient data to extract or calculate prevalence (sample size and number of positive cases), (3) reported the distribution of *Eimeria* species and potential risk factors in cattle and (4) conducted on naturally infected cattle, not experimental infections. Exclusion criteria included review articles, conference abstracts, editorials, case reports, studies without relevant prevalence data or with unclear diagnostic methods or unclear findings and experimental studies without natural epidemiological context.

### 2.4 Data Extraction and Management

Two independent reviewers screened titles and abstracts, followed by full‐text evaluation to determine eligibility. Discrepancies were resolved through discussion or consultation with a third reviewer. Data extracted from eligible studies included first author, publication year, country, continent, sample size, number of positive cases, cattle characteristics (age and sex), season of sampling, quality assessment score, mean annual precipitation, national cattle population, diagnostic method and *Eimeria* species identified.

### 2.5 Quality Assessment

The methodological quality of included studies was assessed using the Joanna Briggs Institute (JBI) critical appraisal checklist (Porritt et al. 2014). The checklist assesses clarity of the research question, study design appropriateness, sample selection adequacy, measurement reliability, control of confounders, completeness of outcome data and statistical analysis appropriateness. On the basis of total scores, studies were categorized as high quality (≥7), moderate quality (4–6.5) or low quality (≤3.5).

### 2.6 Statistical Analysis

Meta‐analysis was performed using comprehensive meta‐analysis (CMA) software version 3 (Brüggemann and Rajguru 2022), with *p* values under 0.05 deemed statistically significant. A random‐effects model was applied to estimate the pooled prevalence of *Eimeria* spp. infection with 95% confidence intervals (CIs), accounting for between‐study variability. Heterogeneity among studies was quantified using the *I* ^2^ statistic, with values above 75% indicating substantial heterogeneity. To explore the pooled prevalence and potential sources of heterogeneity, subgroup analyses were conducted based on: continent, country, year of publication, and sample size. As most studies did not provide denominators for each age and sex group, true subgroup prevalence could not be calculated. Instead, we pooled the proportion of positive cases within each subgroup relative to all positives, using a random‐effects model. These results reflect the distribution of infection among positive animals, not subgroup prevalence in the population. A forest plot was utilized to illustrate the pooled prevalence estimates along with their corresponding 95% CIs, whereas a funnel plot was employed to evaluate the presence of publication bias. Meta‐regression analyses were performed to investigate the influence of continuous covariates including sample size, mean annual precipitation (mm/year) (https://data.worldbank.org/indicator/AG.LND.PRCP.MM), year of publication, and national cattle population (https://worldpopulationreview.com/country‐rankings/cattle‐population‐by‐country) on prevalence estimates. Sensitivity analyses were conducted by sequentially excluding studies to assess the robustness of the pooled estimates. The genetic diversity and species distribution of *Eimeria* spp. in cattle, as well as the seasonal patterns of prevalence, were assessed and presented descriptively. Seasonal data were extracted as reported by each study and categorized into four standard meteorological seasons (spring, summer, autumn and winter) or region‐specific equivalents (e.g., monsoon) when applicable. Because denominators were not consistently provided, seasonal patterns were analysed descriptively by counting the frequency of peak infection periods reported across studies rather than calculating pooled prevalence estimates.

## 3 Results

### 3.1 Study Selection

**FIGURE 1:**
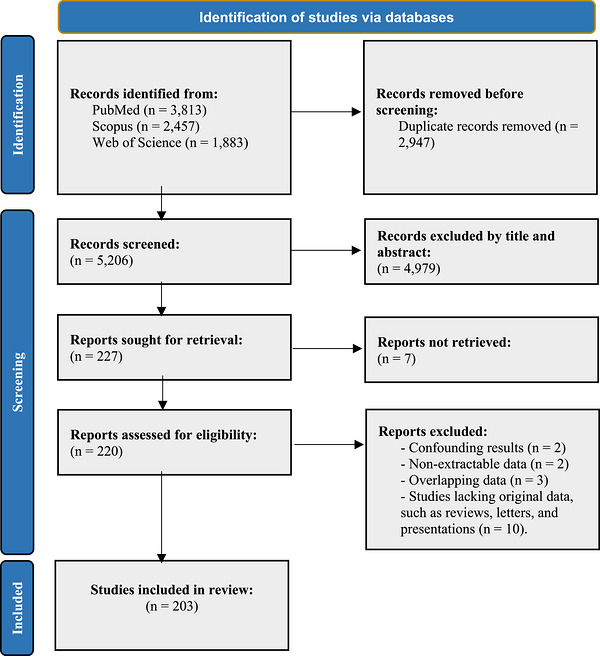
The PRISMA 2020 flow diagram depicting the process of included studies in the present systematic review.

The initial search across international databases yielded 8153 records. After removing 2947 duplicates, 5206 records were screened by title and abstract, of which 4979 were excluded as irrelevant. A total of 227 reports were sought for retrieval. Thus, 220 full‐text articles were assessed for eligibility. Of these, 17 were excluded for reasons including confounding results (*n* = 2), non‐extractable data (*n* = 2), overlapping datasets (*n* = 3) and absence of original data (reviews, letters and presentations; *n* = 10). Finally, 203 studies (Becker and Frye 1929; Sayın 1970; McKenna 1972; Jang 1972; Ruiz 1973; Skandar 1973; Ciordia 1975; Majaro and Dipeolu 1981; Yangxian and Fuqiang 1984; Ernst et al. 1984; Kasim and Al‐Shawa 1985; Musaev et al. 1986; Wee et al. 1988; Munyua and Ngotho 1990; Oda and Nishida 1990; Hasbullah et al. 1990; Hassan and El‐Bahi 1992; Svensson 1993; Rodríguez‐Vivas et al. 1996; Kambarage et al. 1996; Chibunda et al. 1997; Arslan and Tuzer 1998; Tamasaukas and Roa 1998; Himonas et al. 1998; Fayer et al. 2000; Balasubramaniam et al. 2001; Matjila and Penzhorn 2002; Diakou and Papadopoulos 2002; Rinaldi et al. 2004; Epe et al. 2004; Mamatha and ED'Souza 2006; Lucas et al. 2007, 2014; Cheong et al. 2007; Cicek et al. 2007; Farkas et al. 2007; Jiménez et al. 2007; Klockiewicz et al. 2007; Rahmeto et al. 2008; Stewart et al. 2008; Yakhchali and Gholami 2008; Chigure et al. 2008; Gul and Ayaz 2008; Gül et al. 2008; Lassen and Järvis 2009; Lassen et al. 2009; Matsubayashi et al. 2009; Pandit 2009; Pilarczyk, Kołodziejczyk, et al. 2009; Pilarczyk, Pilarczyk, et al. 2009; Al‐Bakry 2009; Díaz et al. 2009, 2010; Gulliksen et al. 2009; Kemper and Henze 2009; Ranka et al. 2010; Lassen 2011; Pilarczyk et al. 2011; Qamar et al. 2011; Rehman et al. 2011; Toaleb et al. 2011; Yu et al. 2011; Davoudi et al. 2011; Almeida Valter dos et al. 2011; Arslan et al. 2012; Marchesi et al. 2012; Bangoura et al. 2012; Singh et al. 2012; Tung et al. 2012; Castagna et al. 2012; Dong et al. 2012; El‐Seify et al. 2012; Alayande et al. 2012; Koutny et al. 2012; Laha et al. 2013; Ntonifor et al. 2013; Nwigwe et al. 2013; Pam et al. 2013; Rusu et al. 2013; Squire et al. 2013, 2019; Enemark et al. 2013; Alemayehu et al. 2013; Murthy and Rao 2014; Ouchene et al. 2014; Tronciu et al. 2014; Heidari et al. 2014; Huang et al. 2014; Ananta et al. 2014; Abbas et al. 2015; Majeed et al. 2015; Peter et al. 2015; Tomczuk et al. 2015, 2018; Yakhchali and Rahmati 2015; Cossío‐Bayúgar et al. 2015; Das et al. 2015; Forslid et al. 2015; Keidāne et al. 2015; Keomoungkhoun and Taweenan 2015; Krishna Murthy and Souza 2016; Kumar et al. 2016, 2021; Marskole et al. 2016; Nath et al. 2016; Takeet et al. 2016; Vanisri et al. 2016; Al‐Jubory and Al‐Rubaie 2016; Haggag et al. 2016; Hamid et al. 2016, 2019; Hiko and Rorisa 2016; Hussin 2016; Andrei et al. 2016; Makau et al. 2017; Ayana et al. 2017; Nechiti et al. 2017; Ayana et al. 2022; Slobodian et al. 2017; Volpato et al. 2017; Coêlho et al. 2017; Dewi et al. 2017; Jamra et al. 2017; Kabir et al. 2017; Kanyari et al. 2017; Adinehbeigi et al. 2018; Lee et al. 2018; Malek and Kuraa 2018; Moussouni et al. 2018; Nehra et al. 2018; Pinilla et al. 2018, 2019; Cardim et al. 2018; Cruvinel et al. 2018, 2020; Dappawar et al. 2018; Diriba and Tulu 2018; Gillandt et al. 2018; Hassoon 2018; Kim et al. 2018; León et al. 2019; Susana et al. 2019; Yusof 2019; Ekawasti et al. 2019, 2021, Ekawasti et al. 2022, 2023; El‐Ashram et al. 2019; Hastutiek et al. 2019; Lopez‐Osorio et al. 2020; Makawi and Abbas 2020; Morgoglione et al. 2020; Nasiri et al. 2020; Ola‐Fadunsin et al. 2020; Ramakrishnan et al. 2020; Sharma and Joshi 2020; Sirbu et al. 2020; Tamrat et al. 2020; Gashaw et al. 2020; Ashour 2021; Pinto et al. 2021; Thanasuwan et al. 2021; Hamdani et al. 2021; Hofer et al. 2021; Ifqiyyah et al. 2021; Karawan and Jarad 2021; Melo et al. 2022, 2023; Nurany et al. 2022; Olivares‐Muñoz et al. 2022; dos Santos et al. 2022; Suleiman et al. 2022; Thapa et al. 2022; Chandra Deb et al. 2022; Gazzonis et al. 2022; Hailu et al. 2022; Hastutiek et al. 2022a, 2022b, 2022c; Jalare et al. 2022; Nurcahyo et al. 2023; Bauer et al. 2023; Terfa et al. 2023; Tiele et al. 2023; Akter et al. 2023; Frias et al. 2023; Guo et al. 2023; Hatam‐Nahavandi et al. 2023; Keomoungkhoun et al. 2023; Mathewos and Endale 2024; Nasrulloh et al. 2024; Pradana et al. 2024; Reshi et al. 2024, 2025; Said and Fahrodi 2024; Srinivasan et al. 2024; Chen et al. 2024; Janah et al. 2024; Kahby et al. 2024; Kamal et al. 2024; López‐Novo et al. 2025; Mengoue et al. 2025; Haryadi et al. 2025) met the inclusion criteria and were included in the systematic review and meta‐analysis (Figure 1).

### 3.2 Study Characteristics and Quality Assessment

A total of 203 studies met the inclusion criteria, representing data from 133,740 cattle across 55 countries and 7 continents. These studies spanned a wide temporal range (1929–2025) and employed microscopy and molecular diagnostic approaches, with most using microscopic examination of faecal samples. The sample size of the studies ranged from 18 to 30,840. Out of the included studies, age‐specific distributions of positive cases were reported in 66 papers/126 datasets, whereas sex‐specific distributions were reported in 34 articles/68 datasets. Cattle were divided into three age groups: calves (under 1 year), young cattle (1–3 years) and adults (over 3 years). Seasonal data were available based on a limited number of studies (*n* = 16) (Table 1). According to the JBI checklist, 25 studies were rated as high quality and 178 studies as moderate quality (Table S1).

**TABLE 1:** Main characteristics of 203 articles included in the current study on the prevalence and distribution of *Eimeria* species in cattle.

Author (year)	Country	Total no.	Infected no.	Prevalence (%)	Method	Season with a high infection rate	Highest prevalence by age group (year)^a^	Highest prevalence by sex
Abbas (2015)	Algeria	690	61	8.8	MIC^b^	UC	UC^c^	UC
Rahmeto et al. (2008)	Ethiopia	580	395	68.1	MIC	UC	<1^*^	UC
Adinehbeigi (2018)	Iran	196	124	63.3	MIC	Spring and Autumn^d^	UC	Male
Akter (2023)	Bangladesh	121	11	9.1	MIC	UC	1–3 and >3^e^	UC
Alayande (2012)	Nigeria	410	145	35.4	MIC	UC	UC	UC
Al‐Bakry (2009)	Iraq	140	36	25.7	MIC	UC	<1	Female
Alemayehu (2013)	Ethiopia	288	92	31.9	MIC	UC	<1^*^	Female
Al‐Jubory (2016)	Iraq	600	211	35.2	MOL^f^	Autumn	1–3	Female
Almeida (2011)	Brazil	117	39	33.3	MIC	UC	<1	UC
Ananta (2014)	Indonesia	394	88	22.4	MIC	UC	UC	UC
Andrei (2016)	Romania	51	10	19.6	MIC	UC	UC	UC
Arslan (1998)	Turkey	768	523	68	MIC	UC	<1	UC
Arslan (2012)	Turkey	125	82	65.6	MIC	UC	UC	UC
Ashour (2021)	Iraq	75	27	36	MIC	UC	UC	UC
Ayana (2017)	Ethiopia	200	110	55	MIC	UC	1–3	Female
Ayana (2022)	Ethiopia	265	119	45	MIC	UC	UC	UC
Balasubramaniam (2001)	India	322	43	13.3	MIC	UC	UC	UC
Bangoura (2012)	Germany	633	376	59.4	MIC	UC	UC	UC
Bauer (2023)	Switzerland	195	84	43.1	MIC	UC	UC	UC
Becker (1929)	USA	40	3	7.5	MIC	UC	UC	UC
Cardim (2018)	Brazil	400	205	51.2	MIC	UC	UC	UC
Castagna (2012)	Italy	500	31	6.2	MIC	UC	UC	UC
Chandra Deb (2022)	Bangladesh	554	308	55.6	MIC	UC	<1^*^	Female
Chen (2024)	China	193	131	67.9	MOL	UC	UC	UC
Cheong (2007)	South Korea	401	157	39.1	MIC	UC	UC	UC
Chibunda (1997)	Tanzania	445	156	35	MIC	UC	<1	UC
Chigure (2008)	India	990	431	43.5	MIC	UC	UC	UC
Cicek (2007)	Turkey	504	163	32.3	MIC	UC	<1	UC
Ciordia (1975)	Georgia	100	71	71	MIC	UC	UC	UC
Coelho (2017)	Brazil	82	44	53.6	MIC	UC	>3	UC
Cossio‐Bayugar (2015)	Mexico	66	6	9.1	MIC	UC	UC	UC
Cruvinel (2020)	Brazil	868	30	3.46	MIC	UC	UC	UC
Cruvinel (2018)	Brazil	2601	667	25.6	MIC	UC	UC	UC
Dappawar (2018)	India	999	13	1.3	MIC	Monsoon	1–3	Female
Das (2015)	India	2339	280	12	MIC	Monsoon	1–3	UC
Davoudi (2011)	Iran	500	90	18	MIC	Summer	<1^*^	UC
Dewi (2017)	Indonesia	130	26	20	MIC	UC	UC	UC
Diakou (2002)	Greece	600	71	11.8	MIC	UC	UC	UC
Diaz (2009)	Spain	904	290	32	MIC	UC	UC	UC
Diaz (2010)	Spain	906	296	32	MIC	UC	>3	UC
Diriba (2018)	Ethiopia	384	184	47.8	MIC	UC	UC	UC
Dong (2012)	China	626	295	47.1	MIC	UC	<1	UC
Dos Santos (2022)	Brazil	387	196	50.6	MIC	UC	<1^*^	UC
Ekawasti (2021)	Indonesia	817	534	65.4	MIC	UC	>3	Male
Ekawasti (2022)	Indonesia	120	36	30	MOL	UC	UC	UC
Ekawasti (2023)	Indonesia	102	59	57.8	MOL	UC	UC	UC
Ekawasti (2019)	Indonesia	289	151	52.3	MIC	UC	>3	UC
El‐Ashram (2019)	Egypt	620	44	7.1	MIC	UC	<1^*^	UC
El‐Seify (2012)	Egypt	698	202	28.9	MIC	Winter	<1^*^	Female
Enemark (2013)	Denmark	453	276	60.9	MIC	UC	UC	UC
Epe (2004)	Germany	998	112	11.2	MIC	UC	UC	UC
Ernst (1984)	Georgia	1809	863	47.7	MIC	UC	UC	UC
Farkas (2007)	Hungary	743	245	33	MIC	UC	UC	UC
Fayer (2000)	USA	163	38	60.3	MIC	UC	UC	UC
Forslid (2016)	Sweden	541	123	23	MIC	UC	UC	UC
Frias (2023)	Peru	1450	577	39.8	MIC	UC	>3	Female
Gashaw (2020)	Ethiopia	378	178	47.1	MIC	UC	>3	Female
Gazzonis (2022)	Italy	495	229	46.2	MIC	UC	UC	UC
Gillandt (2018)	Germany	708	143	20.2	MIC	UC	UC	UC
Gul (2008a)	Turkey	173	96	55.49	MIC	UC	UC	UC
Gul (2008b)	Turkey	182	41	22.53	MIC	UC	UC	UC
Gulliksen (2009)	Norway	759	382	50.3	MIC	UC	UC	UC
Guo (2023)	China	144	12	8.3	MIC	UC	<1^*^	UC
Haggag (2016)	Egypt	100	20	20	MIC	UC	<1	Female
Hailu (2022)	Ethiopia	330	71	21.5	MIC	UC	<1^*^	Female
Hamdani (2021)	Indonesia	100	54	54	MIC	UC	1–3	UC
Hamid (2016)	Indonesia	455	70	15.3	MIC	UC	UC	UC
Hamid (2019)	Indonesia	2150	1510	70.2	MIC	UC	<1	UC
Haryadi (2025)	Indonesia	106	51	48.1	MIC	UC	1–3	UC
Hasbullah (1990)	Japan	2019	390	19.3	MIC	Spring	UC	UC
Hassan (1992)	Egypt	423	42	9.9	MIC	UC	UC	UC
Hasson (2018)	Iraq	100	39	39	MIC	UC	1–3	Female
Hastutiek (2019)	Indonesia	500	267	53.4	MIC	UC	UC	UC
Hastutiek (2022a)	Indonesia	100	21	21	MOL	UC	UC	UC
Hastutiek (2022b)	Indonesia	120	70	58.3	MIC	UC	1–3 and >3	UC
Hastutiek (2022c)	Indonesia	183	50	27.3	MOL	UC	1–3	UC
Hatam‐Nahavandi (2023)	Iran	88	12	13.6	MIC	UC	1–3	Male
Heidari (2014)	Iran	400	33	8.2	MIC	UC	<1	Female
Hiko (2016)	Ethiopia	384	278	72.4	MIC	UC	<1^*^	Female
Himonas (1998)	Macedonia	100	1	1	MIC	UC	UC	UC
Hofer (2021)	Austria	189	53	28	MIC	UC	UC	UC
Huang (2014)	Taiwan	1259	149	11.8	MIC	UC	UC	UC
Hussin (2016)	Iraq	200	19	9.5	MIC	UC	<1	Female
Ifqiyyah (2021)	Indonesia	100	15	15	MIC	UC	UC	UC
Jalare (2022)	India	271	83	30.6	MIC	Summer	<1^*^	Female
Jamra (2017)	India	688	69	10	MIC	Winter	1–3	Female
Janah (2024)	Indonesia	141	63	44.7	MIC	UC	>3	Female
Jang (1972)	South Korea	39	29	74.3	MIC	UC	UC	UC
Jimenez (2007)	Costa Rica	1013	954	94.2	MIC	UC	UC	UC
Kabir (2017)	Bangladesh	109	13	11.9	MIC	UC	UC	UC
Kahby (2024)	Indonesia	21	3	14.3	MIC	UC	UC	UC
Kamal (2024)	Egypt	150	33	22	MIC	UC	UC	UC
Kambarage (2011)	Tanzania	242	107	44.2	MIC	UC	1–3	UC
Kanyari (2010)	Kenya	349	105	30	MIC	UC	UC	UC
Karawan (2021)	Iraq	100	89	89	MOL	UC	UC	UC
Kasim (1985)	Saudi Arabia	205	70	34.1	MIC	UC	UC	UC
Keidane (2015)	Latvia	640	153	23.9	MIC	UC	1–3	UC
Kemper (2009)	Germany	692	204	29.5	MIC	UC	UC	UC
Keomoungkhoun (2015)	Thailand	300	145	48.3	MIC	UC	UC	UC
Keomoungkhoun (2023)	Thailand	296	104	35.1	MOL	UC	<1	UC
Kim (2018)	South Korea	479	124	25.9	MIC	UC	UC	UC
Klockiewicz (2007)	Poland	579	423	73	MIC	UC	UC	UC
Koutny (2012)	Austria	868	727	83.7	MIC	UC	UC	UC
Krishna Murthy (2016)	India	336	90	26.7	MIC	UC	UC	UC
Kumar (2016)	India	88	10	11.4	MIC	UC	UC	UC
Kumar (2021)	India	2469	273	11	MIC	Monsoon	<1	UC
Laha (2013)	India	676	34	5	MIC	UC	UC	UC
Lassen (2009a)	Estonia	887	332	37	MIC	UC	UC	UC
Lassen (2009b)	Lithuania	105	58	55	MIC	UC	UC	UC
Lassen (2011)	Latvia	114	53	46	MIC	UC	UC	UC
Lee (2018)	South Korea	1261	279	22.1	MOL	UC	UC	UC
Leon (2019)	Colombia	103	18	17.4	MIC	UC	1–3	UC
Lopez‐Novo (2025)	Spain	404	28	6.9	MIC	UC	<1^*^	UC
Lopez‐Osorio (2020)	Colombia	1333	1006	75.5	MIC	UC	<1^*^	UC
Lucas (2007)	USA	467	463	99	MIC	UC	UC	UC
Lucas (2014)	USA	827	747	90.3	MIC	UC	UC	UC
Majaro (1981)	Nigeria	2600	1456	56	MIC	UC	UC	UC
Majeed (2015)	Kuwait	86	4	4.6	MIC	UC	UC	UC
Makau (2017)	UK	914	300	32.8	MIC	UC	UC	UC
Makawi (2020)	Iraq	270	114	42.2	MIC	UC	>3	Female
Malek (2018)	Egypt	75	35	46.7	MOL	Summer	<1^*^	UC
Mamatha (2006)	India	500	96	19.2	MIC	UC	UC	UC
Marchesi (2012)	Italy	288	109	37.8	MIC	UC	UC	UC
Marskole (2016)	India	76	21	27.6	MIC	UC	UC	UC
Mathewos (2024)	Ethiopia	460	82	17.8	MIC	Autumn	>3	Female
Matjila (2002)	South Africa	1936	850	44	MIC	UC	UC	UC
Matsubayashi (2009)	Japan	213	163	76.5	MIC	UC	UC	UC
McKenna (1972)	New Zealand	288	153	53	MIC	UC	UC	UC
Melo (2022)	Brazil	800	137	17.2	MIC	UC	UC	UC
Melo (2023)	Brazil	832	307	36.9	MIC	UC	<1	Female
Mengoue (2025)	Cameroon	264	44	16.7	MIC	UC	UC	UC
Morgoglione (2020)	Italy	30,840	28,281	91.7	MIC	UC	UC	UC
Moussouni (2018)	Algeria	143	64	44.7	MIC	UC	1–3	Female
Munyua (1990)	Kenya	620	418	67.4	MIC	UC	UC	UC
Murthy (2014)	India	150	15	10	MIC	UC	UC	UC
Musaev (1987)	Azerbaijan	3389	288	8.5	MIC	UC	UC	UC
Nasiri (2019)	Iran	160	15	9.4	MOL	UC	UC	UC
Nasrulloh (2024)	Indonesia	167	96	57.5	MOL	UC	UC	UC
Nath (2016)	Bangladesh	400	3	0.7	MIC	UC	UC	Male
Nechiti (2017)	Romania	31	10	32.2	MIC	UC	>3	Female
Ntonifor (2013)	Cameroon	277	58	20.9	MIC	UC	UC	UC
Nurany (2022)	Indonesia	62	15	24.2	MIC	UC	UC	Female
Nurcahyo (2023)	Indonesia	171	43	25.1	MOL	UC	1–3	UC
Nwigwe (2013)	Nigeria	569	19	3.3	MIC	UC	UC	UC
Oda (1990)	Japan	1015	599	59	MIC	UC	<1	UC
Ola‐Fadunsin (2020)	Nigeria	478	186	38.9	MIC	UC	1–3	Female
Olivares‐Munoz (2022)	Mexico	930	370	39.7	MIC	UC	UC	UC
Ouchene (2024)	Algeria	690	61	8.8	MIC	UC	<1	UC
Pam (2013)	Nigeria	106	28	26.4	MIC	UC	UC	UC
Pandit (2009)	India	971	711	73.2	MIC	Winter	UC	UC
Peter (2015)	Kenya	110	47	42.7	MIC	UC	UC	UC
Pilarczyk (2009a)	Poland	135	19	14.1	MIC	UC	UC	UC
Pilarczyk (2009b)	Poland	106	19	17.9	MIC	UC	UC	UC
Pilarczyk (2011)	Poland	177	37	20.9	MIC	UC	UC	UC
Pinilla (2018)	Colombia	862	672	77.9	MIC	UC	1‐3	UC
Pinilla (2019)	Colombia	196	38	19.4	MIC	UC	UC	UC
Pinto (2021)	India	726	151	20.8	MIC	UC	UC	UC
Pradana (2024)	Indonesia	300	238	79.3	MIC	UC	1–3	Female
Qamar (2011)	Pakistan	2100	1440	68.6	MIC	UC	UC	UC
Ramakrishnan (2020)	India	86	37	43	MIC	UC	UC	UC
Ranka (2010)	Croatia	641	91	14.2	MIC	UC	UC	UC
Rehman (2011)	Pakistan	584	275	47.1	MIC	UC	>3^*^	Female
Reshi (2024)	India	190	87	45.7	MOL	UC	UC	UC
Reshi (2025)	India	1198	589	49.2	MIC	UC	UC	UC
Rinaldi (2004)	Italy	975	725	74.3	MIC	UC	UC	UC
Rodriguez‐Vivas (1996)	Mexico	1200	1054	87.8	MIC	UC	UC	UC
Ruiz (1973)	USA	823	450	54.7	MIC	UC	UC	UC
Said (2024)	Indonesia	98	27	27.5	MIC	UC	UC	UC
Sayın (1970)	Turkey	150	140	93.3	MIC	UC	UC	UC
Sharma (2020)	India	100	15	15	MIC	Monsoon	<1^*^	Male
Singh (2012)	India	159	6	3.8	MIC	UC	<1^*^	Female
Sirbu (2020)	Romania	303	72	23.7	MIC	UC	>3	UC
Skandar (1973)	Mexico	100	81	81	MIC	UC	UC	UC
Slobodian (2017)	Ukraine	1361	398	29.2	MIC	UC	UC	UC
Squire (2013)	Ghana	309	91	29.4	MIC	UC	1–3	Female
Squire (2019)	Ghana	328	264	80.5	MIC	UC	UC	UC
Srinivasan (2024)	India	90	71	78.9	MIC	UC	UC	UC
Rusu et al. (2013)	Moldova	800	234	29.2	MIC	UC	UC	UC
Stewart (2008)	England^g^	1253	88	7	MIC	Spring	UC	UC
Suleiman (2022)	Nigeria	108	18	16.7	MIC	UC	UC	UC
Susana (2019)	Indonesia	100	53	53	MIC	UC	UC	UC
Nehra et al. (2018)	India	18	6	33.3	MIC	UC	UC	UC
Svensson (1993)	Sweden	429	30	7	MIC	UC	UC	UC
Takeet (2016)	Nigeria	205	195	95.1	MIC	UC	1–3	UC
Tamasaukas (1998)	Venezuela	2145	952	44.4	MIC	UC	UC	UC
Tamrat (2020)	Ethiopia	86	42	48.8	MIC	UC	UC	UC
Terfa (2023)	Ethiopia	691	249	36	MIC	UC	UC	UC
Thanasuwan (2021)	Thailand	330	131	39.7	MIC	UC	UC	UC
Thapa (2022)	Nepal	100	27	27	MIC	UC	UC	UC
Tiele (2023)	Ethiopia	400	11	2.7	MIC	UC	<1	UC
Toaleb (2011)	Egypt	157	138	87.9	MIC	UC	UC	UC
Tomczuk (2018)	Poland	276	68	24.6	MIC	UC	UC	UC
Tomczuk (2015)	Poland	356	188	52.8	MIC	UC	UC	UC
Tronciu (2014)	Moldavia	140	47	33.6	MIC	UC	UC	UC
Tung (2012)	Taiwan	310	37	11.9	MIC	UC	UC	UC
Vanisri (2016)	India	50	8	16	MIC	Monsoon	<1	UC
Volpato (2017)	Brazil	243	53	21.8	MIC	UC	UC	UC
Wee (1988)	South Korea	1092	30	2.7	MIC	UC	UC	UC
Yakhchali (2015)	Iran	307	98	31.9	MIC	UC	UC	UC
Yangxian (1984)	China	372	186	50	MIC	UC	UC	UC
Yu (2011)	China	228	90	39.5	MIC	UC	1–3	UC
Yusof (2019)	Malaysia	152	86	56.6	MIC	UC	UC	UC

*Note*: Because many of the included studies did not report the prevalence of *Eimeria* spp. in cattle stratified by sex or age group, and in several cases the exact sample size of infected and total animals within male, female, and specific age categories was not provided, we reported prevalence descriptively for these variables/subgroups. However, due to the lack of sufficient and precise data, sex‐ and age‐specific prevalence could not be statistically analysed or incorporated as subgroups in the meta‐analysis.

^a^ Cattle were categorized into three age groups based on the purposes of this study: calves younger than 1 year, young cattle between 1 and 3 years and adult cattle older than 3 years. Most of the included studies followed a similar classification system. In cases where studies reported age groups differently, their data were carefully reclassified into these three standardized categories to ensure consistency and comparability across studies. In some studies, the most heavily infected age group was marked with an asterisk (*). This indicates that the cattle included in that study were exclusively from that specific age category, and therefore the reported prevalence values represent only that group.

^b^ MIC: Microscopic detection method.

^c^ UC: Unclear.

^d^ The highest prevalence was observed equally in these two seasons.

^e^ The highest prevalence was observed equally in these two age groups.

^f^ MOL: Molecular detection method.

^g^ In this study, cattle samples were collected from both England and Wales. However, for consistency and to ensure a more coherent analysis, all samples were categorized under England.

### 3.3 Global Prevalence of *Eimeria* spp. in Cattle

The global pooled prevalence of *Eimeria* spp. in cattle was estimated at 33.6% (95% CI: 29.6–37.8) using a random‐effects model. Substantial heterogeneity was detected across studies (*I* ^2^ = 99.4%, *p* < 0.001), justifying the use of subgroup and meta‐regression analyses (Figure S1).

### 3.4 Subgroup Analyses

Subgroup analyses were performed to investigate differences in the pooled prevalence of bovine *Eimeria* spp. infection according to publication year, continent, sample size and country (Table 2). By publication year, pooled prevalence estimates were 40.8% (95% CI: 34.9–47.0) for studies published before 2011, 27.1% (95% CI: 22.5–32.3) for 2012–2018 and 34.3% (95% CI: 26.3–43.2) for 2019–2025. Although point estimates/event rates suggested some temporal variation, heterogeneity within each stratum remained very high (*I* ^2^ > 98%), and thus the differences should be interpreted with caution (Figure S2).

**TABLE 2:** Subgroup analysis of *Eimeria* spp. prevalence in cattle by publication year, country, continent and sample size.

Subgroup variable	Prevalence % (95% CI)	Heterogeneity (*Q*)	No. studies	df (*Q*)	*I* ^2^ (%)	*p* value
**Publication year**						
<2011	40.8 (34.9–47)	7878.3	64	63	99.2	<0.05
2012–2018	27.1 (22.5–32.3)	5236.6	69	68	98.7	<0.05
2019–2025	34.3 (26.3–43.2)	16,640	70	69	99.6	<0.05
**Continent**						
Africa	33.9 (27.8–40.6)	2574.8	40	39	98.5	<0.05
Asia	29.5 (25–34.4)	7274.9	88	87	98.8	<0.05
Central America	67.4 (31.2–90.4)	783.1	5	4	99.5	<0.05
Europe	33.8 (24.1–45.2)	18,803.1	50	49	99.7	<0.05
North America	66.9 (32.3–89.5)	415.9	5	4	99	<0.05
Oceania	53.1 (47.3–58.8)	0	1	0	0	>0.05
South America	36.2 (26–47.9)	1712.8	14	13	99.2	<0.05
**Sample size**						
≤600	33.5 (30.2–36.9)	5463.9	143	142	97.4	<0.05
>600	34.6 (26.7–43.6)	28,322.2	60	59	99.8	<0.05
**Country**						
Algeria	16.4 (5.2–41.1)	120.9	3	2	98.3	<0.05
Austria	58.7 (10.2–94.7)	192.3	2	1	99.5	<0.05
Azerbaijan	8.5 (7.6–9.5)	0	1	0	0	<0.05
Bangladesh	10.1 (1.6–44.2)	171.3	4	3	98.2	<0.05
Brazil	28.6 (19.7–39.6)	463.2	9	8	98.3	<0.05
Cameroon	19.1 (16.4–22.1)	1.6	3	2	0	<0.05
China	40.8 (28.4–54.4)	95.1	5	4	95.8	<0.05
Colombia	58.6 (38.1–76.4)	108.4	3	2	98.1	<0.05
Costa Rica	94.2 (92.6–95.5)	0	1	0	0	<0.05
Croatia	14.2 (11.7–17.1)	0	1	0	0	<0.05
Denmark	60.9 (56.4–65.3)	0	1	0	0	<0.05
Egypt	28.2 (13.7–49.2)	318.3	7	6	98.1	<0.05
Estonia	37.4 (34.3–40.7)	1.3	1	0	0	<0.05
Ethiopia	37.9 (27.2–49.8)	577.6	12	11	98.1	<0.05
Georgia	59.3 (35.7–79.3)	19.2	2	1	94.8	<0.05
Germany	27.2 (11.9–50.9)	410.1	4	3	99.3	<0.05
Ghana	56.7 (12.2–92.5)	149.9	2	1	99.3	<0.05
Greece	11.8 (9.5–14.7)	6.9	1	0	0	<0.05
Hungary	33 (29.7–36.4)	0	1	0	0	<0.05
India	20.8 (13.9–30)	2305.8	23	22	99	<0.05
Indonesia	39.8 (31.1–49.1)	989.2	23	22	97.8	<0.05
Iran	20.6 (11–35.1)	221.4	7	6	97.3	<0.05
Iraq	38.2 (25.7–52.6)	129.4	7	6	95.4	<0.05
Italy	50.7 (18.5–82.3)	2166.1	5	4	99.8	<0.05
Japan	50.8 (19–82)	563.2	3	2	99.6	<0.05
Kenya	46.7 (22.7–72.3)	122.9	3	2	98.4	<0.05
Kuwait	4.7 (1.8–11.7)	0	1	0	0	<0.05
Latvia	34 (16–58.3)	23.6	2	1	95.8	<0.05
Lithuania	55.2 (45.7–64.5)	0	1	0	0	<0.05
Macedonia	1 (0.1–6.8)	1.5	1	0	0	<0.05
Malaysia	56.6 (48.6–64.2)	0	1	0	0	<0.05
Mexico	55.2 (19.5–86.2)	522.1	4	3	99.4	<0.05
Moldova	30 (26.9–100)	1.1	2	1	5.6	<0.05
Nepal	27 (19.2–36.5)	0	1	0	0	<0.05
New Zealand	53.1 (47.3–58.8)	1.2	1	0	0	<0.05
Nigeria	36.3 (21–54.9)	445.7	7	6	98.6	<0.05
Norway	50.3 (46.8–53.9)	0	1	0	0	<0.05
Pakistan	58.3 (36.7–77.1)	88.5	2	1	98.9	<0.05
Peru	39.8 (37.3–42.3)	0	1	0	0	<0.05
Poland	31.8 (15.5–54.2)	317.8	6	5	98.4	<0.05
Romania	24 (20–28.5)	1.7	3	2	0	<0.05
Saudi Arabia	34.1 (28–40.9)	7.8	1	0	0	<0.05
South Africa	43.9 (41.7–46.1)	5.7	1	0	0	<0.05
South Korea	25.1 (12.7–43.6)	258.3	5	4	98.4	<0.05
Spain	21.1 (11.8–34.7)	84.7	3	2	97.6	<0.05
Sweden	13.1 (3.8–36.4)	40.1	2	1	97.5	<0.05
Switzerland	43.1 (36.3–50.1)	0	1	0	0	<0.05
Taiwan	11.9 (10.3–13.5)	2.4	2	1	0	<0.05
Tanzania	39.3 (30.8–48.6)	5.5	2	1	81.9	<0.05
Thailand	41 (33.8–48.6)	11	3	2	81.9	<0.05
Turkey	58.7 (38.9–76.1)	274.6	6	5	98.2	<0.05
UK	16.1 (3–54.5)	202.8	2	1	99.5	<0.05
Ukraine	29.2 (26.9–31.7)	6.8	1	0	0	<0.05
USA	66.9 (32.3–89.5)	415.9	5	4	99	<0.05
Venezuela	44.4 (42.3–46.5)	8.8	1	0	0	<0.05

Abbreviation: CI, confidence interval.

By continent, pooled prevalence ranged from 29.5% (95% CI: 25.0–34.4) in Asia to 67.4% (95% CI: 31.2–90.4) in Central America. The highest pooled prevalence was observed in Central America and North America (66.9%, 95% CI: 32.3–89.5), whereas lower pooled prevalence was reported in Africa (33.9%, 95% CI: 27.8–40.6) and Europe (33.8%, 95% CI: 24.1–45.2). Oceania (53.1%, 95% CI: 47.3–58.8) also showed relatively high prevalence, although only a single study was available from this region (Figure S3).

By sample size, studies with ≤600 animals had a pooled prevalence of 33.5% (95% CI: 30.2–36.9), whereas studies with >600 animals reported 34.6% (95% CI: 26.7–43.6). The difference between groups was small, but both strata exhibited substantial heterogeneity (*I* ^2^ > 97%) (Figure S4).

By country, pooled prevalence estimates varied widely, from as low as 1% in Macedonia (95% CI: 0.1–6.8) and 4.7% in Kuwait (95% CI: 1.8–11.7), to as high as 94.2% in Costa Rica (95% CI: 92.6–95.5). Notably, some country‐level estimates were derived from a single study (Azerbaijan, Costa Rica, Croatia, Denmark, Estonia, Greece, Hungary, Kuwait, Lithuania, Macedonia, Malaysia, Nepal, New Zealand, Norway, Peru, Saudi Arabia, South Africa, Switzerland, Ukraine and Venezuela), whereas other 35 countries were based on multiple reports. The reliability of comparisons across countries is therefore limited, and results should be interpreted cautiously (Figure S5).

### 3.5 Distribution of Positive *Eimeria* spp. in Cattle by Age and Sex

Across 66 studies/126 datasets, calves <1 year accounted for the largest share of positive cases (56.3%, 95% CI: 46.2–65.8), followed by animals aged 1–3 years (45%, 95% CI: 37.2–53.1), whereas adults >3 years (42.7%, 95% CI: 33.8–52.1) contributed a smaller proportion of positives (Table 3 and Figure S6); similarly, female cattle represented the highest proportion of positives (66.7%, 95% CI: 61.7–71.4) in the sex‐based analysis (Table 3 and Figure S7). These pooled proportions reflect the case‐mix among infected animals. These data were therefore analysed separately as proportions of positive cases within each subgroup/variable, rather than as true subgroup prevalence.

**TABLE 3:** Distribution of positive *Eimeria* spp. cases in cattle, categorized by age and sex.

Variables	Prevalence % (95% CI)^a^	No. datasets^b^	df (*Q*)
**Age groups (Y)**			
<1	56.3 (46.2–65.8)	56	55
1–3	45 (37.2–53.1)	46	45
>3	42.7 (33.8–52.1)	24	23
**Sex**			
Female	66.7 (61.7–71.4)	32	31
Male	32.6 (28.1–37.4)	32	31

^a^ Values represent the proportion of positives within each age or sex category relative to the total number of positives, not true subgroup prevalence, as denominators (total tested per category) were not consistently reported.

^b^ The variation in the number of datasets is due to the fact that the evaluated cattle in some studies did not include all three age groups. In certain studies, either all cattle or only positive cases belonged to a specific age group or at most two age groups. This makes it difficult to accurately assess the proportion of positive cases by age group, and therefore the results should be interpreted with caution.

### 3.6 Seasonal Prevalence of *Eimeria* spp. in Cattle

On the basis of the limited number of studies reporting seasonal prevalence (*n* = 16), a higher descriptive frequency of peak *Eimeria* spp. infections was observed during warm and humid periods (monsoon and summer; 8 studies) compared with colder seasons (winter; 3 studies) or transitional periods (spring and autumn; 5 studies) (Table 1).

### 3.7 Species Distribution of *Eimeria* in Cattle

A total of 16 *Eimeria* species were reported in cattle (Table 4): *E. bovis*, *E. zuernii*, *E. auburnensis*, *E. ellipsoidalis*, *E. cylindrica*, *E. alabamensis*, *E. subspherica*, *E. canadensis*, *E. bukidnonensis*, *E. wyomingensis*, *E. brasiliensis*, *E. pellita*, *E. illinoisensis*, *E. smithi*, *E. bombayensis* and *E. ildefonsoi*.

**TABLE 4:** Distribution of *Eimeria* species in cattle based on studies included.

Author (year)	No. *Eimeria* species reported	*Eimeria* species reported^a^	Three most prevalent species
Abbas (2015)	—	*Eimeria* spp.	—
Rahmeto et al. (2008)	11	*E. bovis, E. zuernii, E. auburnensis, E. canadensis, E. ellipsoidalis, E. subspherica, E. cylindrica, E. alabamensis, E. wyomingensis, E. bukidnonensis* and *E. brasiliensis*	*E. bovis, E. zuernii* and *E. auburnensis*
Adinehbeigi (2018)	8	*E. bovis, E. zuernii, E. subspherica, E. brasiliensis, E. ellipsoidalis, E. cylindrica, E. pellita* and *E. wyomingensis*	*E. bovis, E. zuernii* and *E. subspherica*
Akter (2023)	—	*Eimeria* spp.	—
Alayande (2012)	9	*E. auburnensis, E. bovis, E. zuernii, E. subspherica, E. cylindrica, E. ellipsoidalis, E. bukidnonensis, E. illinoisensis* and *E. alabamensis*	*E. auburnensis, E. bovis* and *E. zuernii*
Al‐Bakry (2009)	8	*E. subspherica, E. zuernii, E. bovis, E. alabamensis, E. canadensis, E. bukidnonensis, E. ellipsoidalis* and *E. cylindrica*	*E. subspherica, E. zuernii* and *E. bovis*
Alemayehu (2013)	5	*E. bovis, E. zuernii, E. auburnensis, E. ellipsoidalis, E. alabamensis* and *Eimeria* spp.	*E. bovis, E. zuernii* and *E. auburnensis*
Al‐Jubory (2016)	6	*E. zuernii, E. cylindrica, E. ellipsoidalis, E. bovis, E. auburnensis* and *E. alabamensis*	*E. zuernii, E. cylindrica* and *E. ellipsoidalis*
Almeida (2011)	10	*E. bovis, E. canadensis, E. zuernii, E. ellipsoidalis, E. cylindrica, E. auburnensis, E. brasiliensis, E. bukidnonensis, E. alabamensis* and *E. subspherica*	*E. bovis, E. canadensis* and *E. zuernii*
Ananta (2014)	—	*Eimeria* spp.	—
Andrei (2016)	—	*Eimeria* spp.	—
Arslan (1998)	10	*E. bovis, E. auburnensis, E. zuernii, E. ellipsoidalis, E. canadensis, E. cylindrica, E. supspherica, E. alabamensis, E. bukidnonensis* and *E. brasiliensis*	*E. bovis, E. auburnensis* and *E. zuernii*
Arslan (2012)	10	*E. bovis, E. zuernii, E. auburnensis, E. supspherica, E. canadensis, E. canadensis, E. alabamensis, E. bukidnonensis, E. cylindrica* and *E. brasiliensis*	*E. bovis, E. zuernii* and *E. auburnensis*
Ashour (2021)	—	*Eimeria* spp.	—
Ayana (2017)	—	*Eimeria* spp.	—
Ayana (2022)	7	*E. bovis, E. subspherica, E. wyomingensis, E. zuernii, E. auburnensis, E. alabamensis* and *E. ellipsoidalis*	*E. bovis, E. subspherica* and *E. wyomingensis*
Balasubramaniam (2001)	—	*Eimeria* spp.	—
Bangoura (2012)	2	*E. zuernii* and *E. bovis*	*E. zuernii* and *E. bovis*
Bauer (2023)	—	*Eimeria* spp.	—
Becker (1929)	—	*Eimeria* spp.	—
Cardim (2018)	10	*E. bovis, E. alabamensis, E. zuernii, E. ellipsoidalis, E. auburnensis, E. canadensis, E. cylindrica, E. subspherica, E. bukidnonensis* and *E. brasiliensis*	*E. bovis, E. alabamensis* and *E. zuernii*
Castagna (2012)	—	*Eimeria* spp.	—
Chandra Deb (2022)	4	*E. bovis, E. zuernii, E. alabamensis* and *E. auburnensis*	*E. bovis, E. zuernii* and *E. alabamensis*
Chen (2024)	6	*E. cylindrica, E. bovis, E. auburnensis, E. zuernii, E. wyomingensis* and *E. canadensis*	*E. cylindrica, E. bovis* and *E. auburnensis*
Cheong (2007)	5	*E. bovis, E. zuernii, E. auburnensis, E. bukidnonensis* and *E. subspherica*	*E. bovis, E. zuernii* and *E. auburnensis*
Chibunda (1997)	7	*E. bovis, E. zuernii, E. ellipsoidalis, E. cylindrica, E. auburnensis, E. alabamensis* and *E. subspherica*	*E. bovis, E. zuernii* and *E. ellipsoidalis*
Chigure (2008)^b^	5	*E. bovis, E. zuernii, E. auburnensis, E. cylindrica* and *E. alabamensis*	*E. bovis, E. zuernii* and *E. auburnensis*
Cicek (2007)	10	*E. bovis, E. auburnensis, E. canadensis, E. brasiliensis, E. zuernii, E. bukidnonensis, E. cylindrica, E. ellipsoidalis, E. alabamensis* and *E. illinoisensis*	*E. bovis, E. auburnensis* and *E. canadensis*
Ciordia (1975)	—	*Eimeria* spp.	—
Coelho (2017)	—	*Eimeria* spp.	—
Cossio‐Bayugar (2015)	—	*Eimeria* spp.	—
Cruvinel (2020)	7	*E. bovis, E. alabamensis, E. zuernii, E. cylindrica, E. ellipsoidalis, E. wyomingensis* and *E. canadensis*	*E. bovis, E. alabamensis* and *E. zuernii*
Cruvinel (2018)	12	*E. bovis, E. zuernii, E. brasiliensis, E. canadensis, E. ellipsoidalis, E. wyomingensis, E. cylindrica, E. auburnensis, E. alabamensis, E. subspherica, E. pellita* and *E. bukidnonensis*	*E. bovis, E. zuernii* and *E. brasiliensis*
Dappawar (2018)	—	*Eimeria* spp.	—
Das (2015)	7	*E. bovis, E. zuernii, E. bukidnonensis, E. auburnensis, E. subspherica, E. ellipsoidalis* and *E. alabamensis*	*E. bovis, E. zuernii* and *E. bukidnonensis*
Davoudi (2011)	—	*Eimeria* spp.	—
Dewi (2017)	—	*Eimeria* spp.	—
Diakou (2002)	—	*Eimeria* spp.	—
Diaz (2009)	4	*E. bovis, E. ellipsoidalis, E. wyomingensis* and *E. zuernii*	*E. bovis, E. ellipsoidalis* and *E. wyomingensis*
Diaz (2010)	4	*E. bovis, E. ellipsoidalis, E. wyomingensis* and *E. zuernii*	*E. bovis, E. ellipsoidalis* and *E. wyomingensis*
Diriba (2018)	—	*Eimeria* spp.	—
Dong (2012)	10	*E. ellipsoidalis, E. bovis, E. zuernii, E. alabamensis, E. auburnensis, E. cylindrica, E. subspherica, E. pellita, E. wyomingensis* and *E. brasiliensis*	*E. ellipsoidalis, E. bovis* and *E. zuernii*
Dos Santos (2022)	11	*E. bovis, E. zuernii, E. ellipsoidalis, E. cylindrica, E. subspherica, E. auburnensis, E. alabamensis, E. canadensis, E. bukidnonensis, E. wyomingensis* and *E. brasiliensis*	*E. bovis, E. zuernii* and *E. ellipsoidalis*
Ekawasti (2021)	—	*Eimeria* spp.	—
Ekawasti (2022)	6	*E. bovis, E. zuernii, E. cylindrica, E. auburnensis, E. alabamensis* and *E. ellipsoidalis*	*E. bovis, E. zuernii* and *E. cylindrica*
Ekawasti (2023)	1	*E. zuernii*	E. zuernii
Ekawasti (2019)	6	*E. bovis, E. ellipsoidalis, E. alabamensis, E. zuernii, E. auburnensis* and *E. cylindrica*	*E. bovis, E. ellipsoidalis* and *E. alabamensis*
El‐Ashram (2019)	—	*Eimeria* spp.	—
El‐Seify (2012)	11	*E. ellipsoidalis, E. cylindrica, E. bovis, E. zuernii, E. subspherica, E. alabamensis, E. canadensis, E. auburnensis, E. wyomingensis, E. brasiliensis* and *E. bukidnonensis*	*E. ellipsoidalis, E. cylindrica* and *E. bovis*
Enemark (2013)	12	*E. ellipsoidalis, E. zuernii, E. bovis, E. cylindrica, E. auburnensis, E. canadensis, E. subspherica, E. alabamensis, E. bukidnonensis, E. wyomingensis, E. pellita* and *E. brasiliensis*	*E. ellipsoidalis, E. zuernii* and *E. bovis*
Epe (2004)	—	*Eimeria* spp.	—
Ernst (1984)	1	*E. bovis*	*E. bovis*
Farkas (2007)	7	*E. auburnensis, E. ellipsoidalis, E. bovis, E. zuernii, E. cylindrica, E. pellita* and *E. subspherica*	*E. auburnensis, E. ellipsoidalis* and *E. bovis*
Fayer (2000)	2	*E. bovis* and *E. ellipsoidalis*	*E. bovis* and *E. ellipsoidalis*
Forslid (2016)	11	*E. bovis, E. zuernii, E. ellipsoidalis, E. auburnensis, E. wyomingensis, E. canadensis, E. cylindrica, E. subspherica, E. alabamensis, E. pellita* and *E. brasiliensis*	*E. bovis, E. zuernii* and *E. ellipsoidalis*
Frias (2023)	—	*Eimeria* spp.	—
Gashaw (2020)	—	*Eimeria* spp.	—
Gazzonis (2022)	—	*Eimeria* spp.	—
Gillandt (2018)	—	*Eimeria* spp.	—
Gul (2008a)	10	*E. bovis, E. auburnensis, E. zuernii, E. cylindrica, E. brasiliensis, E. alabamensis, E. subspherica, E. bukidnonensis, E. canadensis* and *E. ellipsoidalis*	*E. bovis, E. auburnensis* and *E. zuernii*
Gul (2008b)	—	*Eimeria* spp.	—
Gulliksen (2009)	—	*Eimeria* spp.	—
Guo (2023)	—	*Eimeria* spp.	—
Haggag (2016)	—	*Eimeria* spp.	—
Hailu (2022)	—	*Eimeria* spp.	—
Hamdani (2021)	—	*Eimeria* spp.	—
Hamid (2016)^b^	6	*E. bovis, E. auburnensis, E. bukidnonensis, E. canadensis, E. zuernii* and *E. cylindrica*	*E. bovis, E. auburnensis* and *E. bukidnonensis*
Hamid (2019)	8	*E. bovis, E. zuernii, E. auburnensis, E. canadensis, E. cylindrica, E. bukidnonensis, E. alabamensis* and *E. ellipsoidalis*	*E. bovis, E. zuernii* and *E. auburnensis*
Haryadi (2025)	8	*E. bovis, E. alabamensis, E. cylindrica, E. zuernii, E. auburnensis, E. wyomingensis, E. pellita* and *E. canadensis*	*E. bovis, E. alabamensis* and *E. cylindrica*
Hasbullah (1990)	13	*E. bovis, E. auburnensis, E. canadensis, E. alabamensis, E. ellipsoidalis, E. zuernii, E. bukidnonensis, E. brasiliensis, E. wyomingensis, E. subspherica, E. cylindrica, E. illinoisensis* and *E. pellita*	*E. bovis, E. auburnensis* and *E. canadensis*
Hassan (1992)	4	*E. bovis, E. subspherica, E. ellipsoidalis* and *E. zuernii*	*E. bovis, E. subspherica* and *E. ellipsoidalis*
Hasson (2018)	—	*Eimeria* spp.	—
Hastutiek (2019)	—	*Eimeria* spp.	—
Hastutiek (2022a)	4	*E. bovis, E. zuernii, E. auburnensis* and *E. cylindrica*	*E. bovis, E. zuernii* and *E. auburnensis*
Hastutiek (2022b)	6	*E. bovis, E. zuernii, E. ellipsoidalis, E. auburnensis, E. subspherica* and *E. canadensis*	*E. bovis, E. zuernii* and *E. ellipsoidal*
Hastutiek (2022c)	4	*E. bovis, E. zuernii, E. auburnensis* and *E. cylindrica*	*E. bovis, E. zuernii* and *E. auburnensis*
Hatam‐Nahavandi (2023)	—	*Eimeria* spp.	—
Heidari (2014)	9	*E. bovis, E. zuernii, E. canadensis, E. ellipsoidalis, E. alabamensis, E. pellita, E. auburnensis, E. cylindrica* and *E. bukidnonensis*	*E. bovis, E. zuernii* and *E. canadensis*
Hiko (2016)	10	*E. bovis, E. zuernii, E. auburnensis, E. canadensis, E. subspherica, E. ellipsoidalis, E. cylindrica, E. alabamensis, E. wyomingensis* and *E. bukidnonensis*	*E. bovis, E. zuernii* and *E. auburnensis*
Himonas (1998)	—	*Eimeria* spp.	—
Hofer (2021)	—	*Eimeria* spp.	—
Huang (2014)	—	*Eimeria* spp.	—
Hussin (2016)	—	*Eimeria* spp.	—
Ifqiyyah (2021)	—	*Eimeria* spp.	—
Jalare (2022).	—	*Eimeria* spp.	—
Jamra (2017)	—	*Eimeria* spp.	—
Janah (2024)	—	*Eimeria* spp.	—
Jang (1972)	6	*E. zuernii, E. ellipsoidalis, E. cylindrica, E. subspherica, E. bovis* and *E. bukidnonensis*	*E. zuernii, E. ellipsoidalis* and *E. cylindrica*
Jimenez (2007)	—	*Eimeria* spp.	—
Kabir (2017)	—	*E. bovis*	*E. bovis*
Kahby (2024)	—	*Eimeria* spp.	—
Kamal (2024)	—	*Eimeria* spp.	—
Kambarage (2011)	—	*Eimeria* spp.	—
Kanyari (2010)	—	*Eimeria* spp.	—
Karawan (2021)	—	*Eimeria* spp.	—
Kasim (1985)	7	*E. zuernii, E. bovis, E. ellipsoidalis, E. cylindrica, E. auburnensis, E. subspherica* and *E. wyomingensis*	*E. zuernii, E. bovis* and *E. ellipsoidalis*
Keidane (2015)	—	*Eimeria* spp.	—
Kemper (2009)	—	*Eimeria* spp.	—
Keomoungkhoun (2015)	—	*E. bovis*	*E. bovis*
Keomoungkhoun (2023)	5	*E. bovis, E. zuernii, E. alabamensis, E. ellipsoidalis* and *E. cylindrica*	*E. bovis, E. zuernii* and *E. alabamensis*
Kim (2018)	5	*E. zuernii, E. auburnensis, E. bovis, E. subspherica* and *E. bukidnonensis*	*E. zuernii, E. auburnensis* and *E. bovis*
Klockiewicz (2007)	12	*E. bovis, E. ellipsoidalis, E. zuernii, E. auburnensis, E. cylindrica, E. alabamensis, E. subspherica, E. canadensis, E. pellita, E. bukidnonensis, E. wyomingensis* and *E. brasiliensis*	*E. bovis, E. ellipsoidalis* and *E. zuernii*
Koutny (2012)	11	*E. bovis, E. ellipsoidalis, E. zuernii, E. auburnensis, E. alabamensis, E. cylindrica, E. canadensis, E. subspherica, E. bukidnonensis, E. pellita* and *E. wyomingensis*	*E. bovis, E. ellipsoidalis* and *E. zuernii*
Krishna Murthy (2016)	—	*Eimeria* spp.	*—*
Kumar (2016)	—	*Eimeria* spp.	*—*
Kumar (2021)	9	*E. zuernii, E. bovis, E. brasiliensis, E. subspherica, E. auburnensis, E. alabamensis, E. bukidnonensis, E. ellipsoidalis* and *E. wyomingensis*	*E. zuernii, E. bovis* and *E. brasiliensis*
Laha (2013)	—	*Eimeria* spp.	*—*
Lassen (2009a)	12	*E. bovis, E. zuernii, E. ellipsoidalis, E. canadensis, E. subspherica, E. auburnensis, E. alabamensis, E. wyomingensis, E. cylindrica, E. brasiliensis, E. pellita* and *E. bukidnonensis*	*E. bovis, E. zuernii* and *E. ellipsoidalis*
Lassen (2009b)	11	*E. bovis, E. zuernii, E. auburnensis, E. alabamensis, E. canadensis, E. ellipsoidalis, E. cylindrica, E. subspherica, E. brasiliensis, E. pellita* and *E. wyomingensis*	*E. bovis, E. zuernii* and *E. auburnensis*
Lassen (2011)	8	*E. zuernii, E. bovis, E. ellipsoidalis, E. alabamensis, E. subspherica, E. cylindrica, E. canadensis* and *E. auburnensis*	*E. zuernii, E. bovis* and *E. ellipsoidalis*
Lee (2018)	3	*E. bovis, E. zuernii* and *E. auburnensis*	*E. bovis, E. zuernii* and *E. auburnensis*
Leon (2019)	—	*Eimeria* spp.	*—*
Lopez‐Novo (2025)	—	*E. bovis* and *E. zuernii*	*E. bovis* and *E. zuernii*
Lopez‐Osorio (2020)	13	*E. bovis, E. auburnensis, E. zuernii, E. pellita, E. ellipsoidalis, E. canadensis, E. wyomingensis, E. bukidnonensis, E. brasiliensis, E. alabamensis, E. subspherica, E. cylindrica* and *E. illinoisensis*	*E. bovis, E. auburnensis* and *E. zuernii*
Lucas (2007)	12	*E. bovis, E. zuernii, E. cylindrica/ellipsoidalis, E. alabamensis, E. canadensis, E. auburnensis, E. illinoisensis, E. pellita, E. subspherica, E. bukidnonensis, E. brasiliensis* and *E. wyomingensis*	*E. bovis, E. zuernii* and *E. cylindrica/ellipsoidalis*
Lucas (2014)	12	*E. bovis, E. zuernii, E. cylindrica/ellipsoidalis, E. auburnensis, E. alabamensis, E. canadensis, E. illinoisensis, E. pellita, E. subspherica, E. brasiliensis, E. wyomingensis* and *E. bukidnonensis*	*E. bovis, E. zuernii* and *E. cylindrica/ellipsoidalis*
Majaro (1981)	9	*E. bovis, E. zuernii, E. auburnensis, E. bukidnonensis, E. ellipsoidalis, E. alabamensis, E. canadensis, E. subspherica* and *E. cylindrica*	*E. bovis, E. zuernii* and *E. auburnensis*
Majeed (2015)	—	*Eimeria* spp.	*—*
Makau (2017)	—	*Eimeria* spp.	*—*
Makawi (2020)	5	*Eimeria* spp.	*—*
Malek (2018)	8	*E. zuernii, E. bovis, E. alabamensis, E. cylindrica, E. subspherica, E. canadensis, E. ellipsoidalis* and *E. auburnensis*	*E. zuernii, E. bovis* and *E. alabamensis*
Mamatha (2006)	—	*Eimeria* spp.	*—*
Marchesi (2012)	7	*E. bovis, E. alabamensis, E. auburnensis, E. canadensis, E. zuernii, E. bukidnonensis* and *E. cylindrica*	*E. bovis, E. alabamensis* and *E. auburnensis*
Marskole (2016)	—	*Eimeria* spp.	*E. bovis* and *E. zuernii*
Mathewos (2024)	2	*E. bovis* and *E. zuernii*	*—*
Matjila (2002)	12	*E. zuernii, E. bovis, E. ellipsoidalis, E alabamensis, E. auburnensis, E. pellita, E. cylindrica, E. subspherica, E. brasiliensis, E. canadensis, E. bukidnonensis* and *E. illinoisensis*	*E. zuernii, E. bovis* and *E. ellipsoidalis*
Matsubayashi (2009)	—	*Eimeria* spp.	*—*
McKenna (1972)	10	*E. bovis, E. zuernii, E. canadensis, E. ellipsoidalis, E. auburnensis, E. alabamensis, E. cylindrica, E. brasiliensis, E. wyomingensis* and *E. subspherica*	*E. bovis, E. zuernii* and *E. canadensis*
Melo (2022)	14	*E. bovis, E. canadensis, E. auburnensis, E. ellipsoidalis, E. zuernii, E. brasiliensis, E. bukidnonensis, E. illinoisensis, E. wyomingensis, E. alabamensis, E. cylindrica, E. pellita, E. ildefonsoi* and *E. subspherica*	*E. bovis, E. canadensis* and *E. auburnensis*
Melo (2023)	—	*Eimeria* spp.	*—*
Mengoue (2025)	—	*Eimeria* spp.	*—*
Morgoglione (2020)	9	*E. subspherica, E. zuernii, E. ellipsoidalis, E. cylindrica, E. alabamensis, E. bovis, E. canadensis, E. wyomingensis* and *E. auburnensis*	*E. subspherica, E. zuernii* and *E. ellipsoidalis*
Moussouni (2018)	—	*Eimeria* spp.	*—*
Munyua (1990)	8	*E. bovis, E. zuernii, E. auburnensis, E. ellipsoidalis, E. cylindrica, E. subspherica, E. wyomingensis* and *E. alabamensis*	*E. bovis, E. zuernii* and *E. auburnensis*
Murthy (2014)	1	*E. zuernii*	*E. zuernii*
Musaev (1987)^b^	9	*E. auburnensis, E. bovis, E. bukidnonensis, E. ellipsoidalis, E. smithi, E. zuernii, E. brasiliensis, E. canadensis* and *E. subspherica*	*E. auburnensis, E. bovis* and *E. bukidnonensis*
Nasiri (2019)	3	*E. bovis, E. auburnensis* and *E. zuernii*	*E. bovis, E. auburnensis* and *E. zuernii*
Nasrulloh (2024)	1	*E. bovis*	*E. bovis*
Nath (2016)	—	*Eimeria* spp.	*—*
Nechiti (2017)	—	*Eimeria* spp.	*—*
Ntonifor (2013)	—	*Eimeria* spp.	*—*
Nurany (2022)	—	*Eimeria* spp.	*—*
Nurcahyo (2023)	4	*E. bovis, E. auburnensis, E. zuernii* and *E. alabamensis*	*E. bovis, E. auburnensis* and *E. zuernii*
Nwigwe (2013)	—	*Eimeria* spp.	*—*
Oda (1990)	11	*E. bovis, E. ellipsoidalis, E. auburnensis, E. brasiliensis, E. cylindrica, E. canadensis, E. alabamensis, E. zuernii, E. wyomingensis, E. bukidnonensis* and *E. subspherica*	*E. bovis, E. ellipsoidalis* and *E. auburnensis*
Ola‐Fadunsin (2020)	8	*E. bovis, E. zuernii, E. auburnensis, E. cylindrica, E. subspherica, E. canadensis, E. bukidnonensis* and *E. alabamensis*	*E. bovis, E. zuernii* and *E. auburnensis*
Olivares‐Munoz (2022)	10	*E. canadensis, E. bovis, E. zuernii, E. cylindrica, E. ellipsoidalis, E. auburnensis, E. wyomingensis, E. alabamensis, E. bukidnonensis* and *E. subspherica*	*E. canadensis, E. bovis* and *E. zuernii*
Ouchene (2024)	—	*Eimeria* spp.	*—*
Pam (2013)	—	*Eimeria* spp.	*—*
Pandit (2009)	—	*Eimeria* spp.	*—*
Peter (2015)	—	*Eimeria* spp.	*—*
Pilarczyk (2009a)	5	*E. bovis, E. auburnensis, E. ellipsoidalis, E. zuernii* and *E. subspherica*	*E. bovis, E. auburnensis* and *E. ellipsoidalis*
Pilarczyk (2009b)	6	*E. bovis, E. zuernii, E. ellipsoidalis, E. subspherica, E. auburnensis* and *E. brasiliensis*	*E. bovis, E. zuernii* and *E. ellipsoidalis*
Pilarczyk (2011)^b^	8	*E. bovis, E. auburnensis, E. ellipsoidalis, E. subspherica, E. zuernii, E. canadensis, E. cylindrica* and *E. alabamensis*	*E. bovis, E. auburnensis* and *E. ellipsoidalis*
Pinilla (2018)	—	*Eimeria* spp.	*—*
Pinilla (2019)	—	*Eimeria* spp.	*—*
Pinto (2021)	—	*Eimeria* spp.	*—*
Pradana (2024)	6	*E. bovis, E. auburnensis, E. zuernii, E. alabamensis, E. ellipsoidalis* and *E. canadensis*	*E. bovis, E. auburnensis* and *E. zuernii*
Qamar (2011)	5	*E. bovis, E. ellipsoidalis, E. zuernii, E. cylindrica* and *E. subspherica*	*E. bovis, E. ellipsoidalis* and *E. zuernii*
Ramakrishnan (2020)	3	*E. alabamensis, E. cylindrica* and *E. bovis*	*E. alabamensis, E. cylindrica* and *E. bovis*
Ranka (2010)	2	*E. bovis* and *E. zuernii*	*E. bovis* and *E. zuernii*
Rehman (2011)	6	*E. bovis, E. zuernii, E. canadensis, E. ellipsoidalis, E. alabamensis* and *E. cylindrica*	*E. bovis, E. zuernii* and *E. canadensis*
Reshi (2024)^b^	3	*E. zuernii, E. alabamensis* and *E. bovis*	*E. zuernii, E. alabamensis* and*E. bovis*
Reshi (2025)^b^	9	*E. zuernii, E. bovis, E. alabamensis, E. auburnensis, E. canadensis, E. subspherica, E. ellipsoidalis, E. cylindrica* and *E. brasiliensis*	*E. zuernii, E. bovis* and*E. alabamensis*
Rinaldi (2004)	10	*E. bovis, E. zuernii, E. ellipsoidalis, E. wyomingensis, E. canadensis, E. cylindrica, E. subspherica, E. auburnensis, E. alabamensis* and *E. pellita*	*E. bovis, E. zuernii* and *E. ellipsoidalis*
Rodriguez‐Vivas (1996)	9	*E. bovis, E. auburnensis, E. ellipsoidalis, E. canadensis, E. zuernii, E. alabamensis, E. cylindrica, E. subspherica* and *E. bukidnonensis*	*E. bovis, E. auburnensis* and *E. ellipsoidalis*
Ruiz (1973)	8	*E. alabamensis, E. canadensis, E. auburnensis, E. subspherica, E. zuernii, E. ellipsoidalis, E. bukidnonensis* and *E. cylindrica*	*E. alabamensis, E. canadensis* and *E. auburnensis*
Said (2024)	—	*Eimeria* spp.	*—*
Sayın (1970)	11	*E. bovis, E. auburnensis, E. ellipsoidalis, E. zuernii, E. canadensis, E. cylindrica, E. bukidnonensis, E. subspherica, E. brasiliensis, E. alabamensis* and *E. illinoisensis*	*E. bovis, E. auburnensis* and *E. ellipsoidalis*
Sharma (2020)	—	*Eimeria* spp.	*—*
Singh (2012)	—	*Eimeria* spp.	*—*
Sirbu (2020)	—	*Eimeria* spp.	*—*
Skandar (1973)	3	*E. bovis, E. auburnensis* and *E. zuernii*	*E. bovis, E. auburnensis* and *E. zuernii*
Slobodian (2017)	6	*E. bovis, E. ellipsoidalis, E. аuburnensis, E. zuernii, E. wyomingensis* and *E. bukidnonensis*	*E. bovis, E. ellipsoidalis* and *E. аuburnensis*
Squire (2013)	—	*Eimeria* spp.	*—*
Squire (2019)	*—*	*Eimeria* spp.	*—*
Srinivasan (2024)	*—*	*Eimeria* spp.	*—*
Rusu et al. (2013)	*—*	*Eimeria* spp.	*—*
Stewart (2008)	12	*E. bovis, E. zuernii, E. ellipsoidalis, E. canadensis, E. alabamensis, E. auburnensis, E. subspherica, E. brasiliensis, E. wyomingensis, E. cylindrica, E. pellita* and *E. bukidnonensis*	*E. bovis, E. zuernii* and *E. ellipsoidalis*
Suleiman (2022)	—	*Eimeria* spp.	*—*
Susana (2019)	—	*Eimeria* spp.	*—*
Nehra et al. (2018)	—	*Eimeria* spp.	*—*
Svensson (1993)^b^	4	*E. alabamensis, E. bovis, E. subspherica* and *E. zuernii*	*E. alabamensis, E. bovis* and *E. subspherica*
Takeet (2016)	—	*Eimeria* spp.	*—*
Tamasaukas (1998)^b^	6	*E. alabamensis, E. bovis, E. subspherica/E. zuernii, E. cylindrica, E. wyomingensis* and *E. auburnensis*	*E. alabamensis, E. bovis* and *E. subspherica/E. zuernii*
Tamrat (2020)	9	*E. bovis, E. zuernii, E. auburnensis, E. canadensis, E. ellipsoidalis, E. subspherica, E. cylindrica, E. alabamensis* and *E. brasiliensis*	*E. bovis, E. zuernii* and *E. auburnensis*
Terfa (2023)	—	*Eimeria* spp.	*—*
Thanasuwan (2021)	—	*Eimeria* spp.	*—*
Thapa et al. (2022)	—	*Eimeria* spp.	*—*
Tiele (2023)	—	*Eimeria* spp.	*—*
Toaleb (2011)	—	*Eimeria* spp.	*—*
Tomczuk (2018)	—	*Eimeria* spp.	*—*
Tomczuk (2015)	8	*E. bovis, E. zuernii, E. canadensis, E. bukidnonensis, E. alabamensis, E. ellipsoidalis, E. auburnensis* and *E. wyomingensis*	*E. bovis, E. zuernii* and *E. canadensis*
Tronciu (2014)	—	*Eimeria* spp.	*—*
Tung (2012)	—	*Eimeria* spp.	*—*
Vanisri (2016)	—	*Eimeria* spp.	*—*
Volpato (2017)	—	*Eimeria* spp.	*—*
Wee (1988)	2	*E bukidnonensis* and *E. wyomingensis*	*E bukidnonensis* and *E. wyomingensis*
Yakhchali (2015)	7	*E. zuernii, E. bovis, E. cylindrica, E. subspherica, E. ellipsoidalis, E. bukidnonensis* and *E. wyomingensis*	*E. zuernii, E. bovis* and *E. cylindrica*
Yakhchali (2008)	8	*E. wyomingensis, E. zuernii, E. cylindrica, E. illinoisensis, E. subspherica, E. alabamensis, E. auburnensis* and *E. canadensis*	*E. wyomingensis, E. zuernii* and *E. cylindrica*
Yangxian (1984)	—	*Eimeria* spp.	
Yu (2011)^b^	12	*E. subspherica, E. ellipsoidalis, E. zuernii, E. pellita, E. bovis, E. canadensis, E. bukidnonensis, E. cylindrica, E. auburnensis, E. brasiliensis, E. bombayensis* and *E. alabamensis*	*E. subspherica, E. ellipsoidalis* and *E. zuernii*
Yusof (2019)	—	*Eimeria* spp.	*—*

^a^ In this column, the reported *Eimeria* species from cattle are presented in descending order, from the highest to the lowest prevalence.

^b^ Studies marked with an asterisk indicate limited data regarding prevalence and species categorization. In these studies, species were not listed in order of prevalence, and species‐specific prevalence remains unclear; therefore, their results should be interpreted with caution.

The descriptive evaluation of species distribution among studies revealed that the most frequently reported species were *E. bovis*, *E. zuernii*, *E. auburnensis*, *E. ellipsoidalis*, *E. cylindrica* and *E. alabamensis*. Additional species, such as *E. subspherica*, *E. canadensis*, *E. bukidnonensis* and *E. wyomingensis*, were also reported relatively frequently. Less common species included *E. brasiliensis*, *E. pellita* and *E. illinoisensis*, whereas three species (*E. smithi*, *E. bombayensis* and *E. ildefonsoi*) were reported only once.

### 3.8 Sensitivity Analyses and Meta‐Regression

The sensitivity analysis, performed by sequentially removing each study and re‐estimating the pooled effect size, showed that no single study markedly altered the overall prevalence estimate of *Eimeria* spp. in cattle. The results remained consistent across iterations, indicating the robustness and stability of the pooled findings (Figure S8).

**FIGURE 2:**
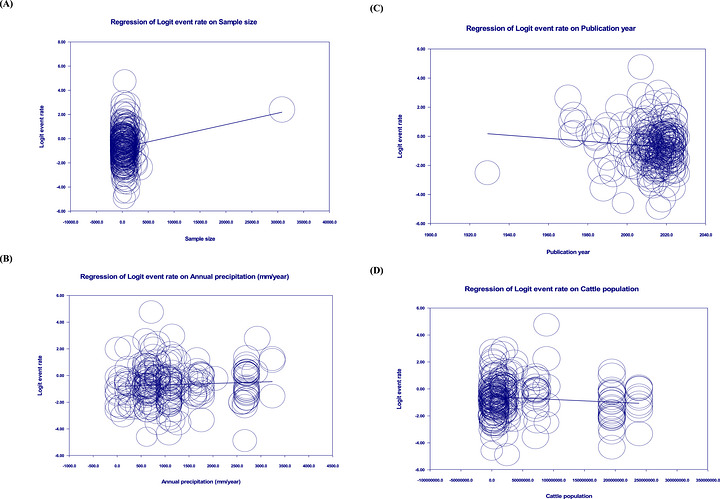
Bubble plots illustrating meta‐regression analyses assessing the effect of (A) sample size, (B) mean annual precipitation, (C) publication year, and (D) national cattle population on the prevalence of bovine *Eimeria* infection. In each plot, the size of the bubbles is proportional to the weight of the study in the meta‐analysis. The fitted regression line represents the relationship between the covariate and the reported prevalence. A significant positive association was observed for sample size (*p* = 0.003), suggesting that larger studies tended to report higher prevalence estimates. In contrast, mean annual precipitation (*p* = 0.45), publication year (*p* = 0.17) and national cattle population (*p* = 0.11) showed no statistically significant effects, indicating that these variables did not explain between‐study heterogeneity.

Meta‐regression analyses were conducted to assess the influence of sample size, mean annual precipitation, year of publication, and national cattle population on the prevalence of bovine *Eimeria* spp. (Figure 2). Among these, only sample size demonstrated a statistically significant association (*β* = 0.0001 and *p* = 0.0038), indicating that larger studies tended to report higher prevalence estimates. Inclusion of sample size into the model reduced between‐study variance (*τ* ^2^) from 1.7526 to 1.0488 and explained approximately 4% of the observed heterogeneity (*R* ^2^ analogue = 0.04), although substantial heterogeneity persisted (*I* ^2^ = 98.93%). In contrast, mean annual precipitation (*β* = 0.0001 and *p* = 0.345), publication year (*β* = −0.0103 and *p* = 0.1724) and national cattle population (*β* ≈ −0.0000 and *p* = 0.111) were not significantly associated with prevalence. These variables did not meaningfully reduce between‐study variance or *I* ^2^. Overall, the analyses suggest that while sample size may account for a small fraction of heterogeneity, the remaining covariates did not significantly influence infection prevalence at the global level, and high residual heterogeneity remained unexplained across all models.

### 3.9 Publication Bias

**FIGURE 3:**
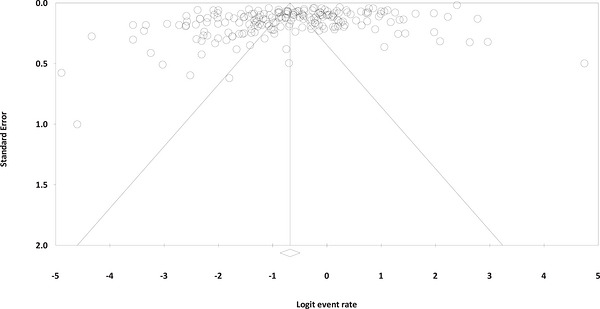
The funnel plot of the included studies assessing the publication bias in the current systematic review and meta‐analysis.

The funnel plot of standard error by logit event rate for the 203 included studies demonstrated a generally symmetrical distribution of effect sizes around the pooled estimate, although slight asymmetry was noted among studies with smaller sample sizes and larger standard errors. Egger's regression intercept test confirmed the presence of small‐study effects, with a statistically significant deviation from symmetry (intercept = −10.41, SE = 1.53, *t* = 6.79, *p* < 0.05). Although this indicates some degree of publication bias, the overall pattern of the funnel plot suggests that the extent of bias is limited and unlikely to materially alter the pooled prevalence estimate (Figure 3).

## 4 Discussion

The present systematic review and meta‐analysis estimated the global pooled prevalence of *Eimeria* spp. infection in cattle at 33.6% (95% CI: 29.6–37.8), underscoring the substantial worldwide burden of bovine coccidiosis. This finding highlights the significance of *Eimeria* spp. as one of the most widespread protozoan parasites impacting cattle health and productivity. When compared with meta‐analyses conducted in other livestock species, the prevalence observed in cattle appears relatively lower. For example, a recent global meta‐analysis of *Eimeria* spp. in goats reported a prevalence of 62.9% (95% CI: 58.6–67.2) (Ali et al. 2025), whereas regional studies in water buffalo, goats and sheep demonstrated even higher infection levels at 92.7% (95% CI: 0.9–100), 75.6% (95% CI: 53.8–89.2) and 47% (95% CI: 30.7–63.9), respectively (Disfani et al. 2025). These differences may reflect host‐specific susceptibility, husbandry and management practices or ecological and climatic factors that differentially influence oocyst transmission and survival across species (Disfani et al. 2025). Nevertheless, the detection of *Eimeria* spp. in approximately one‐third of cattle worldwide confirms that bovine coccidiosis remains an endemic and economically significant disease, warranting sustained attention from veterinary researchers, livestock producers and animal health policymakers.

The descriptive global evaluation reveals that *E. bovis* and *E. zuernii* are the most widely distributed and frequently reported *Eimeria* species infecting cattle, a finding that aligns with their recognized role as the primary pathogenic agents of clinical coccidiosis in calves (Bangoura et al. 2012; Forslid et al. 2015). Together with *E. auburnensis*, *E. ellipsoidalis*, *E. cylindrica* and *E. alabamensis*, these species constitute the ‘core group’ of *Eimeria* in cattle herds worldwide. Some species, such as *E. subspherica*, *E. canadensis*, *E. bukidnonensis* and *E. wyomingensis*, were also reported relatively frequently, whereas species, such as *E. wyomingensis*, *E. bukidnonensis* and *E. pellita*, showed intermediate frequencies, reflecting potential geographical specificity, differences in management systems (intensive housing vs. grazing), age susceptibility or diagnostic sensitivity (Ali et al. 2025; Disfani et al. 2025). Rare species, including *E. smithi* (Musaev et al. 1986), *E. bombayensis* (Yu et al. 2011) and *E. ildefonsoi* (Melo et al. 2022), were recorded in only single studies and may represent regionally restricted taxa, host‐specific variants or possibly taxonomic misidentifications due to morphological similarity. From a methodological perspective, this study assessed presence/absence of species across studies rather than quantitative prevalence estimates for each species. Consequently, these results should be interpreted as descriptive evidence of global distribution patterns, not as definitive measures of the relative burden of infection. Many earlier studies relied primarily on morphological identification of oocysts, which is inherently limited by overlapping size ranges and morphometric similarities (e.g., between *E. cylindrica* and *E. ellipsoidalis*) (Lucas et al. 2014). Advances in molecular diagnostics, such as PCR and DNA barcoding, are expected to refine species‐level identification and reduce misclassification in future epidemiological research.

When compared with other ruminants, a similar epidemiological pattern emerges: A limited number of pathogenic species dominate the clinical burden, whereas numerous other species occur sporadically or as co‐infections. For example, in sheep and goats, Eimeria *ovinoidalis* and Eimeria *arloingi*/*E. ninakohlyakimovae* are recognized as the most pathogenic species (Chartier and Paraud 2012; Ali et al. 2025), mirroring the dominance of *E. bovis* and *E. zuernii* in cattle. This reinforces the importance of focusing control measures and preventive strategies on the most clinically relevant species, while acknowledging the ecological value of understanding the broader species distribution. Finally, interpretation of species distribution should be cautious because in several countries the data were derived from only a single study, whereas in others (e.g., India, Indonesia, Ethiopia and Brazil) multiple reports were available. Such imbalances reduce the reliability of direct cross‐country comparisons. Moreover, ‘top three dominant species’ entries in some studies may not reflect true prevalence in the underlying population but instead may be influenced by study design, season or age group. Despite these limitations, the synthesis provides the most comprehensive overview to date of the global distribution of *Eimeria* species in cattle and highlights the key species of epidemiological and clinical significance.

The subgroup analyses highlight marked variation in reported prevalence of *Eimeria* spp. in cattle across time, continents, and countries. Although pooled prevalence estimates differed between publication periods, no consistent trend of increase or decline was evident, and high within‐group heterogeneity indicates that temporal differences cannot be reliably interpreted as true epidemiological changes. Similarly, the variation observed across continents and countries underscores the potential influence of ecological, climatic and management factors, but the interpretation is complicated by the uneven distribution of studies. For example, high prevalence estimates in Central and North America were based on relatively few studies, whereas estimates for Asia and Africa reflected a larger number of reports. At the national level, extremely high or low prevalence values in countries represented by a single study may reflect study‐specific conditions (e.g., herd management, diagnostic method and sampling season) rather than true national prevalence. In contrast, countries with numerous studies, such as India, Indonesia, Ethiopia and Brazil, provide more stable estimates, though substantial heterogeneity remained even within these strata. Differences in diagnostic techniques, sampling strategies and herd management practices likely contributed to the observed variability and should be considered when comparing results across regions.

With regard to sample size, although studies with larger sample sizes reported slightly higher prevalence, the difference was modest, and heterogeneity persisted in both subgroups. This suggests that methodological and epidemiological diversity among studies plays a greater role than sample size alone in shaping prevalence estimates. Taken together, the subgroup analyses suggest that geographic, temporal and methodological factors may influence prevalence patterns, but the substantial residual heterogeneity indicates that unmeasured factors such as breed susceptibility, intensity of management, biosecurity practices and climatic micro‐conditions likely contribute to the global variability of *Eimeria* spp. prevalence in cattle. These results should therefore be interpreted with caution, particularly when comparing estimates from countries with limited or single‐study data.

The descriptive analysis of seasonal distribution highlights a potential influence of climatic and environmental factors on the prevalence of *Eimeria* spp. infections in cattle. The observation that several studies have identified the monsoon and summer seasons as periods of highest prevalence is biologically plausible, as high humidity and warm temperatures can favour the sporulation and survival of oocysts in the environment (Ekawasti et al. 2021). Reports of peak infections in other seasons (spring, autumn or winter) may reflect regional climatic differences, variations in cattle management practices or methodological heterogeneity across studies. Nonetheless, the interpretation of these findings is limited by the small number of studies reporting seasonal data (16 of 203), the lack of standardized definitions of seasons across geographic regions and the absence of consistent quantitative measures of prevalence by season. These limitations hinder the ability to draw firm conclusions or to generalize patterns globally. Future epidemiological studies should report seasonal prevalence data more systematically, ideally alongside relevant environmental and management variables, to better elucidate the role of seasonality in the epidemiology of bovine coccidiosis.

In the present analysis, the age distribution of positive cases revealed that calves younger than 1 year harboured the highest proportion of infections (56.3%, 95% CI: 46.2–65.8), followed by young cattle aged 1–3 years (45.0%, 95% CI: 37.2–53.1). Adult animals older than 3 years exhibited comparatively lower proportions of infection (42.7%, 95% CI: 33.8–52.1). A similar pattern was observed in the sex‐related data, where female cattle accounted for the majority of positive cases (66.7%, 95% CI: 61.7–71.4). These findings suggest that susceptibility to *Eimeria* spp. infection is greatest in younger animals, which is consistent with their immunological immaturity and lack of prior exposure to the parasite. The relatively lower infection proportions observed in adults may reflect the gradual development of acquired immunity following repeated subclinical exposures (Matos et al. 2018). The higher proportion of positives among females could be influenced by management‐related factors, such as longer retention of females for breeding purposes and greater exposure to contaminated environments compared to males, which are often slaughtered earlier. Taken together, these results emphasize the importance of targeted preventive strategies in young cattle and breeding females, as these groups appear to contribute disproportionately to the overall burden of *Eimeria* spp. infections in cattle populations. Of note, age and sex patterns derived from the composition of positive cases should be interpreted with caution, as they do not reflect true subgroup prevalence without appropriate denominators.

The results of the meta‐regression highlight that heterogeneity in the prevalence of bovine *Eimeria* spp. infection cannot be sufficiently explained by broad ecological or temporal covariates. Sample size was the only significant predictor, with larger studies more likely to detect and report higher prevalence, consistent with the notion that small studies may underestimate infection rates due to limited representation and higher random error. However, the effect size was modest and explained only 4% of the variability, underscoring the need to consider methodological rigour and sample adequacy in interpreting prevalence data. Of note, the finding that the sample size was statistically significant but explained only 4% of the heterogeneity suggests that study precision contributed modestly to variability in reported prevalence. Larger studies may capture a broader representation of management systems, production types and environmental conditions, which can naturally lead to higher detected prevalence. However, the small proportion of explained heterogeneity indicates that sample size alone does not account for the substantial methodological and epidemiological diversity present across studies. The lack of significant associations for mean annual precipitation and publication year suggests that neither large‐scale climatic indicators nor temporal changes in study conduct systematically influenced prevalence estimates. Although rainfall and humidity are known to affect oocyst survival and sporulation (Ekawasti et al. 2021), their impact may be masked at the global level by overriding factors such as farm‐level hygiene, management practices and regional husbandry systems. Similarly, the non‐significant association between national cattle population and infection prevalence reflects the limited explanatory power of aggregated country‐level data, which may obscure within‐country heterogeneity in production systems and biosecurity practices. Taken together, these findings emphasize that the observed high heterogeneity (*I* ^2^ > 99%) is likely attributable to unmeasured study‐level and regional variables, including diagnostic methods, breed susceptibility and management conditions, rather than to ecological or temporal factors captured in this analysis. Future research should prioritize standardized methodologies and detailed reporting of management and environmental conditions to better elucidate the drivers of variability in *Eimeria* spp. prevalence across cattle populations.

The sensitivity analysis confirmed the robustness of the meta‐analysis findings. Excluding individual studies did not substantially change the direction or magnitude of the pooled prevalence estimate. This stability suggests that the overall results are not driven by any single study and strengthens the validity and reliability of the conclusions regarding the prevalence and distribution of *Eimeria* spp. in cattle.

The assessment of publication bias revealed a modest degree of asymmetry, particularly among smaller studies, as reflected in both the funnel plot and the highly significant Egger's regression test. Such asymmetry may indicate selective publication of studies with higher prevalence estimates or reflect methodological heterogeneity among smaller datasets. However, the relatively symmetrical distribution of larger studies around the pooled estimate suggests that the global prevalence derived in this meta‐analysis remains robust. The sensitivity analysis further confirmed that exclusion of individual studies did not materially affect the overall results. Taken together, these findings imply that although publication bias cannot be entirely excluded, its influence on the global prevalence estimate of *Eimeria* spp. in cattle is likely minimal, supporting the reliability and validity of the present conclusions.

Although the present study represents a comprehensive systematic review and meta‐analysis of the global prevalence and distribution of *Eimeria* species in cattle, several limitations should be acknowledged. First, as in most meta‐analyses, the availability and quality of primary data restricted the scope of our analyses. In some subgroup categories (e.g., specific continents or countries), the pooled prevalence estimates were based on a limited number of studies, and in several cases, only a single study contributed to the estimate. Such imbalances reduce the reliability of cross‐group comparisons and necessitate cautious interpretation (Shams et al. 2022). Second, despite extensive subgroup and meta‐regression analyses, the substantial heterogeneity observed across studies remained largely unexplained, suggesting that important sources of variability such as breed susceptibility, farm‐level management practices or environmental micro‐conditions were not consistently reported and thus could not be incorporated. Third, although species‐level data on *Eimeria* spp. were available in some studies, inconsistent reporting formats and incomplete information prevented quantitative synthesis at the species level; therefore, species distribution was summarized descriptively rather than through pooled prevalence estimates. Finally, variability in diagnostic methods, study design and sample size across the included studies likely contributed additional bias and residual heterogeneity that could not be fully addressed. Taken together, these limitations highlight the need for more standardized, large‐scale epidemiological investigations that systematically report both overall prevalence and species‐level data across diverse geographic and production contexts.

Prevention of *Eimeria* spp. infections in cattle relies primarily on improving farm management and hygiene, including regular removal of faecal material, maintaining dry and clean housing conditions, avoiding overcrowding and ensuring adequate ventilation. Strategic use of anticoccidial drugs and coccidiosis vaccines, where available, may also reduce the risk of infection and clinical outbreaks, particularly in young calves. The findings of this global meta‐analysis provide valuable epidemiological insights that can inform veterinary practitioners, livestock producers and policymakers. Moreover, they may guide national and international organizations such as the Food and Agriculture Organization (FAO), the World Organisation for Animal Health (WOAH) and agricultural extension services in developing evidence‐based strategies for coccidiosis control and prevention in cattle populations worldwide.

## 5 Conclusion

This systematic review and meta‐analysis, by pooling data from 203 studies encompassing more than 133,000 cattle across 55 countries, demonstrates that *Eimeria* spp. infections remain endemic in cattle worldwide, with a pooled prevalence of 33.6%. The most frequently reported and clinically important species were *E. bovis* and *E. zuernii*, whereas several additional species occurred sporadically or regionally with variable frequency. Considerable heterogeneity across studies persisted despite subgroup and meta‐regression analyses, suggesting that management practices, diagnostic approaches and host‐related factors play a greater role than broad ecological or temporal variables. Although subject to limitations such as uneven study distribution and descriptive species‐level data, this work highlights the substantial veterinary and economic impact of bovine coccidiosis and provides evidence to guide control efforts and policy development at both national and international levels.

## Author Contributions

Ali Asghari and Laya Shamsi planned and designed the study. Ali Pouryousef, Mohammad Reza Mohammadi, Milad Badri, Mostafa Omidian and Oskar Nowak were involved in the methodology and data extraction. Ali Asghari and Laya Shamsi conducted the statistical analysis. Ali Asghari, Laya Shamsi and Ali Pouryousef wrote the manuscript and revised it. All authors have read and approved the final manuscript.

## Funding

The authors have nothing to report.

## Ethics Statement

This study was based exclusively on previously published data and did not involve the collection of new samples from animals. Therefore, no additional ethical approval was required. The study protocol, including the use of secondary data, was reviewed and approved by the Ethics Committee of Khoy University of Medical Sciences, Khoy, Iran (approval no. IR.KHOY.REC.1404.039).

## Conflicts of Interest

The authors declare no conflicts of interest.

## Supporting information




**Supporting Table 1**: JBI critical appraisal checklist applied for included studies.

**Supporting Figure 1**: Forest plot showing the prevalence of *Eimeria* spp. in cattle. Each horizontal black line represents the 95% confidence interval (CI) for the prevalence reported in an individual study. The red circles indicate the point estimate (event rate) for each study. The vertical solid line at 0 represents the null value (no events), whereas the vertical dashed line represents the overall pooled prevalence estimated from the random‐effects model. The diamond at the bottom summarizes the pooled prevalence and its 95% CI.

**Supporting Figure 2**: The pooled prevalence of *Eimeria* spp. in cattle based on publication year. Red indicates the prevalence from each study, whereas grey shows the overall weighted prevalence.

**Supporting Figure 3**: The pooled prevalence of *Eimeria* spp. in cattle based on continent. Red indicates the prevalence from each study, whereas grey shows the overall weighted prevalence.

**Supporting Figure 4**: The pooled prevalence of *Eimeria* spp. in cattle based on sample size. Red indicates the prevalence from each study, whereas grey shows the overall weighted prevalence.

**Supporting Figure 5**: The pooled prevalence of *Eimeria* spp. in cattle based on country. Red indicates the prevalence from each study, whereas grey shows the overall weighted prevalence.

**Supporting Figure 6**: Pooled prevalence and distribution of positive *Eimeria* spp. in cattle by age.

**Supporting Figure 7**: Pooled prevalence and distribution of positive *Eimeria* spp. in cattle by sex.

**Supporting Figure 8**: Sensitivity analysis assessing the influence of individual studies on pooled estimates.

## Data Availability

The datasets used and/or analysed during the current study are available in the online version.
