# Supplementary material for: Eimeria spp. in Cattle: A Global Systematic Review and Meta‐Analysis
Source: Vet Med Sci. 2026 May 11;12(3):e70991. doi: 10.1002/vms3.70991 (PMC13159717; doi:10.1002/vms3.70991)
Supplement: Supplementary file 2 — Supporting Figure 1: Forest plot showing the prevalence of Eimeria spp. in cattle. Each horizontal black line represents the 95% confidence interval (CI) for the prevalence reported in an individual study. The red circles indicate the point estimate (event rate) for each study. The vertical solid line at 0 represents the null value (no events), whereas the vertical dashed line represents the overall pooled prevalence estimated from the random‐effects model. The diamond at the bottom summarizes the pooled prevalence and its 95% CI. [file VMS3-12-e70991-s006.docx]

**Supplementary Fig. 1.** Forest plot showing the prevalence of *Eimeria* spp. in cattle. Each horizontal black line represents the 95% confidence interval (CI) for the prevalence reported in an individual study. The red circles indicate the point estimate (event rate) for each study. The vertical solid line at 0 represents the null value (no events), while the vertical dashed line represents the overall pooled prevalence estimated from the random-effects model. The diamond at the bottom summarizes the pooled prevalence and its 95% CI.
