# Supplementary material for: Eimeria spp. in Cattle: A Global Systematic Review and Meta‐Analysis
Source: Vet Med Sci. 2026 May 11;12(3):e70991. doi: 10.1002/vms3.70991 (PMC13159717; doi:10.1002/vms3.70991)
Supplement: Supplementary file 5 — Supporting Figure 4: The pooled prevalence of Eimeria spp. in cattle based on sample size. Red indicates the prevalence from each study, whereas grey shows the overall weighted prevalence. [file VMS3-12-e70991-s004.docx]

**Supplementary Fig. 4.** The pooled prevalence of *Eimeria* spp. in cattle based on sample size. Red indicates the prevalence from each study, while grey shows the overall weighted prevalence.
