# Supplementary material for: Eimeria spp. in Cattle: A Global Systematic Review and Meta‐Analysis
Source: Vet Med Sci. 2026 May 11;12(3):e70991. doi: 10.1002/vms3.70991 (PMC13159717; doi:10.1002/vms3.70991)
Supplement: Supplementary file 7 — Supporting Figure 6: Pooled prevalence and distribution of positive Eimeria spp. in cattle by age. [file VMS3-12-e70991-s005.docx]

**Supplementary Fig. 6.** Pooled prevalence and distribution of positive *Eimeria* spp. in cattle by Age.
