# Supplementary material for: Eimeria spp. in Cattle: A Global Systematic Review and Meta‐Analysis
Source: Vet Med Sci. 2026 May 11;12(3):e70991. doi: 10.1002/vms3.70991 (PMC13159717; doi:10.1002/vms3.70991)
Supplement: Supplementary file 9 — Supporting Figure 8: Sensitivity analysis assessing the influence of individual studies on pooled estimates. [file VMS3-12-e70991-s001.docx]

**Supplementary Fig. 8.** Sensitivity analysis assessing the influence of individual studies on pooled estimates.
